# Human Ebola virus infection in West Africa: a review of available therapeutic agents that target different steps of the life cycle of Ebola virus

**DOI:** 10.1186/2049-9957-3-43

**Published:** 2014-11-28

**Authors:** Kang Yiu Lai, Wing Yiu George Ng, Fan Fanny Cheng

**Affiliations:** Department of Intensive Care, Queen Elizabeth Hospital, HKSAR, B6, 30 Gascoigne Rd, Kowloon, Hong Kong SAR China; Department of Medicine, Queen Elizabeth Hospital, HKSAR, Kowloon, Hong Kong SARChina

**Keywords:** Ebola virus, Non-mutable host cell therapeutic targets for Ebola virus, Cocktail therapeutic intervention for RNA virus

## Abstract

The recent outbreak of the human Zaire ebolavirus (EBOV) epidemic is spiraling out of control in West Africa. Human EBOV hemorrhagic fever has a case fatality rate of up to 90%. The EBOV is classified as a biosafety level 4 pathogen and is considered a category A agent of bioterrorism by Centers for Disease Control and Prevention, with no approved therapies and vaccines available for its treatment apart from supportive care. Although several promising therapeutic agents and vaccines against EBOV are undergoing the Phase I human trial, the current epidemic might be outpacing the speed at which drugs and vaccines can be produced. Like all viruses, the EBOV largely relies on host cell factors and physiological processes for its entry, replication, and egress. We have reviewed currently available therapeutic agents that have been shown to be effective in suppressing the proliferation of the EBOV in cell cultures or animal studies. Most of the therapeutic agents in this review are directed against non-mutable targets of the host, which is independent of viral mutation. These medications are approved by the Food and Drug Administration (FDA) for the treatment of other diseases. They are available and stockpileable for immediate use. They may also have a complementary role to those therapeutic agents under development that are directed against the mutable targets of the EBOV.

**Electronic supplementary material:**

The online version of this article (doi:10.1186/2049-9957-3-43) contains supplementary material, which is available to authorized users.

## Multilingual abstract

Please see Additional file 1 for translations of the abstract into the six official working languages of the United Nations.

## Background

The recent outbreak of the human Zaire ebolavirus (EBOV) infection starting in West African countries has resulted in 15,351 infected patients, as of 18^th^ of November 2014. A total of 5,459 deaths have been reported in six affected countries (Guinea, Liberia, Mali, Sierra Leone, Spain, and the United States of America) and two previously affected countries (Nigeria and Senegal) [1]. Apart from supportive care, neither a licensed vaccine nor a specific therapy is available for the treatment of the human EBOV infection [2]. The World Health Organization (WHO) has considered that it is ethically acceptable to offer unproven interventions that have shown promising results in laboratory and animal models, but have not yet been evaluated for safety and efficacy in humans as potential sources of treatment or prevention [3]. Several promising therapeutic agents have been identified for the treatment and immunization of the EBOV. These may include monoclonal antibody (mAbs)-based therapies (e.g. ZMapp), anti-sense phosphorodiamidate morpholino oligomers (PMO AVI-6002), lipid nanoparticle small interfering RNA (LNP-siRNA: TKM-Ebola), and an EBOV glycoprotein-based vaccine using live-attenuated recombinant vesicular stomatitis virus (rVSV-EBOGP) or a chimpanzee adenovirus (rChAd-EBOGP)-based vector. Human trial results of these agents would not be available until next year. Moreover, existing supplies of all these experimental medications and vaccines for compassionate use are either extremely limited or exhausted [4–6]. To combat such an unprecedented global public-health crisis before these experimental agents are available, alternative available interventions that can target different steps in the replication cycle of the EBOV should be explored in the management of the human EBOV infection as contingency preparation for the international dissemination of the EBOV outbreak in West Africa. We have reviewed currently available therapeutic agents that have shown to be effective in suppressing the proliferation of the EBOV in cell cultures or animal studies. We propose a therapeutic regimen to supplement the current supportive therapy aiming to reduce viral load, the most important factor in the determination of mortality. Through viral load suppression, we may be able to prolong a patient’s survival in order to provide a better chance for the patient to develop natural immune defense against the EBOV.

## Discussion

### The genome of the Ebola virus

The EBOV is an enveloped filamentous RNA virus belonging to the family *Filoviridae*. The 19-kb linear, non-segmented, negative-sense, single-stranded RNA genome of the EBOV encodes seven structural proteins and two non-structural proteins in the following order within the genome: 3′ non-coding region (leader), nucleoprotein (NP), virion protein 35 (VP35), VP40, 3 glycoproteins (sGP/ssGP/GP1,2), VP30, VP24, RNA-dependent RNA-polymerase protein (L-polymerase), and 5′ non-coding region [7].

### The glycoproteins of the Ebola virus

The EBOV genome encodes one transmembrane protein GP1,2 (GP1–GP2) and two secreted non-structural proteins: secretary glycoprotein (sGP) and small soluble glycoprotein (ssGP). A small soluble delta peptide (Δ-peptide) is secreted from EBOV-infected cells after the carboxyl-terminal cleavage of sGP [8]. GP1,2 is produced through transcriptional RNA editing as a precursor for 676 amino acid polyprotein (GP0), which is post-translationally cleaved by furin into two disulfide-linked subunits; a surface subunit, GP1; and a membrane-spanning subunit, GP2. GP1 contains the receptor-binding domain (RBD) for host cell attachment and a mucin-like domain to protect the RBD from humoral and cell-mediated immunity. The RBD responsible for receptor binding, viral entry, and cellular tropism is covered by a heavily glycosylated “glycan cap”. The transmembrane GP2 contains a helical heptad-repeat region, transmembrane anchor, and a 4-residue cytoplasmic tail. The GP2 drives fusion of the viral membrane with the endosomal membrane of the target cell. This GP1–GP2 heterodimer then assembles as a trimer on the viral surface. This homotrimeric GP1,2 complex forms the spike on the envelope membrane of the mature viral particles. During processing, GP1,2 are unstable, and an abundant amount of a soluble non-virion form of GP1 and a scanty amount of GP1,2 are released into the circulation [9–12]. The virus-associated GP1,2 and not the other soluble glycoproteins released during the virus infection are responsible for primary target cell activation [13]. The highly glycosylated mucin-like region of GP1 is cytotoxic to the host cells [14]. The shedding of souble GP1,2-like protein due to cleavage of EBOV glycoprotein on the surface of EBOV-infected cells by tumor necrosis factor-alpha converting enzyme (TACE) can activate non-infected dendritic cells and macrophages to induce cytokine dysregulation and endothelial cell dysfunction [15]. The GP2 of the EBOV is able to counter the interferon (IFN)-inducible antiviral protein tetherin which restricts the VP40-dependent budding of the progeny viral particles from infected cells [16–18]. The sGP is produced from non-edited mRNA species through furin cleavage from a precursor pre-sGP. The sGP shares the N-terminal 295 amino acids with GP1, but differs in the carboxyl terminus by 69 amino acids. The sGP is released into the circulation in the form of homodimers in anti-parallel orientation [19] to evade an antibody-associated innate immune response [20, 21]. The sGP has an anti-inflammatory function and impairs the transmigration and activation of neutrophils [22, 23]. While the GP1,2 in its particle-associated form mediates endothelial cell activation and a decrease in endothelial cell barrier function, sGP protects the endothelial cell against cytokine-induced barrier dysfunction. The sGP constitutes at greater than 80% of the total GP synthesized during infection. Hence, the hypersecretion of the sGP may protect the EBOV against host humoral immune defense and the host endothelial cell against cytokine-induced cytotoxicity during the early phase of the EBOV infection [15, 24, 25]. Δ-peptide released in EBOV-infected cells joins cathepsins and integrins to inhibit further entry of the EBOV in a dose-dependent manner to prevent superinfection of EBOV-infected cells. Δ-peptide inhibits entry of both marburgviruses and the EBOV, indicating that they might interfere with a common pathway used by filoviruses to gain entry into target cells [26]. The ssGP of a yet undefined function is produced through transcriptional editing and secreted in the form of a disulfide-linked homodimer that is exclusively N-glycosylated. While ssGP appears to share similar structural properties with sGP, it does not appear to have the same anti-inflammatory function as sGP [22, 23, 27].

### The life cycle of the Ebola virus

**Figure 1:**
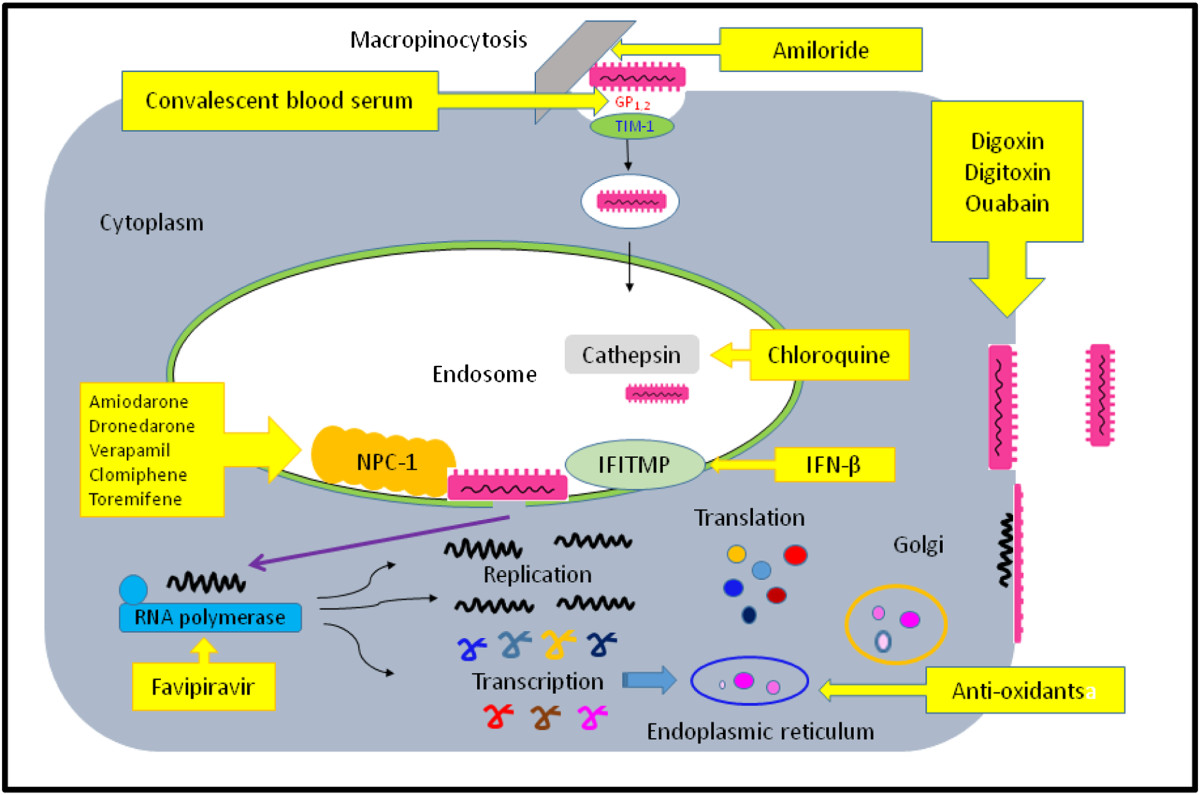
**Schematic diagram showing the replication cycle of Ebola virus (EBOV):** Upon receptor binding of EBOV GP_1_ with host TIM-1 receptor, EBOV is internalized into endosome via macropinocytosis. Within the acidified endosome compartment of the host cell, under the action of the low pH-dependent cellular proteases cathepsins, the receptor binding site of GP_1_ to cholesterol transporter Niemann-Pick C1 (NPC1) is exposed. This results in conformational change in GP_2_ , leading to complete fusion of the viral and host endosomal membranes in the late endosome and the release of viral RNA and its associated proteins into the host cell cytoplasm. EBOV then hijacks transcription and translation for robust genome replication and viral protein production under the action of ribonucleoprotein polymerase complex (RNP polymerase). The accumulation of GP_1,2_ in the endoplasmic reticulum leads to endoplasmic reticulum overload response (ER-overload) which, in turn, induces cytokine dysregulation via the activation of nuclear factor kappa B (NFκB) through the production of reactive oxygen species (ROS). New virions are released through ATP-dependent budding and egress from host cell membrane. Currently available therapeutic agents that target the different steps of the EBOV life cycle are described in Table 1.

The EBOV, being a RNA virus with limited coding capacity, has utilized the host’s unique metabolic pathway for its viral entry, replication, and egress. The entry of the EBOV into cells is initiated by interaction of the viral GP1 with host cell surface T-cell immunoglobulin and mucin domain 1 (TIM-1) receptors. Upon receptor binding, the EBOV is internalized into endosomes primarily via macropinocytosis [28–30]. Within the acidified endosome compartment of the host cell, the heavily glycosylated GP1 is cleaved to a smaller 19-kDa fusogenic form by the low pH-dependent cellular proteases Cathepsin L (CatL) and B (CatB), exposing residues in the receptor binding site. This allows the binding of GP1 to cholesterol transporter Niemann-Pick C1 (NPC1), a step in the late endosome phase essential for virus-host membrane fusion and viral entry [31–34]. Cells where the NPC1 function has been biochemically disrupted or cells lacking NPC1 showed resistance to the EBOV infection. Cells from subjects with NPC1 disease were resistant to the EBOV because of defects in the NPC1 protein [35–38]. After complete fusion of the viral and host endosomal membranes via conformational change in GP2, viral RNA and its associated proteins are released into the host cell cytoplasm [39]. Once inside the cytoplasm of the host cell, the EBOV suppresses the innate immune response via VP35 and VP24 proteins [40], and hijacks transcription and translation for robust genome replication and the production of new virions. The ribonucleoprotein (RNP) complex that mediates transcription and replication of the EBOV genome comprises NP, VP35, VP30, and L protein [41–44]. VP30 is essential in the initiation of the EBOV transcription, but is not required for viral replication. However, dynamic phosphorylation of VP30 is an important mechanism to regulate the balance between the transcription and replication processes in the EBOV replication cycle [45–47]. This unique property of VP30 allows the development of a genetically stable VP30 deleted EBOV vaccine with protective efficacy in the mice and guinea pig models [48]. The matrix proteins VP40 and VP24 associated with the viral lipid coat are important for virus structure and stability. Both matrix proteins VP24 and VP40 contribute to the regulation of viral genome replication and transcription [49] and the budding of the virus [50–52], an important step prior to viral egress [53, 54]. This distinct replication cycle of the EBOV serves as an attractive target for the development of therapeutic agents against the EBOV (see Figure 1 and Table 1).

**Table 1:** **Available therapeutic agents that target the different steps of the EBOV life cycle as shown in the diagram**

Medication	Mechanism of action
Convalescent blood serum	Contain neutralizing antibodies to provide passive immunity.
Na^+^/K^+^ exchanger	Inhibit virus uptake by macropinocytosis.
- Amiloride	
Chloroquine^1^	Leads to alkalinization of endosomes and prevent the acid pH-dependent cleavage of Ebola virus GP_1,2_ by endosomal proteases cathepsin B and L.
Cationic amphiphiles	Induce a Niemann-Pick C-like phenotype and block the entry of EBOV through late endosomes.
Amiodarone^1^	
Dronedarone^1^	
Verapamil^2^	
Clomiphene	
Toremifene^1^	
Interferon- beta (IFN-β)	Induce interferon-inducible transmembrane proteins (IFITMP) production to restrict entry of EBOV.
Favipiravir	Suppress viral RNA polymerase.
Na+/K+/ATPase pump inhibitors	Inhibit Na^+^/K^+^-ATPase that are important in the budding and egress of encapsulated EBOV.
Ouabain	
Digoxin	
Digitoxin	
Anti-oxidants	Suppress ROS-dependent NFκB activation and cytokine dysregulation induced by GP_1,2_-induced ER-overload.
High dose N-acetylcysteine infusion	

^1^Chloroquine, Amiodarone, Dronedarone and Toremifene administration is associated with an increased risk of QT prolongation and *Torsades de pointes*. ^2^Verapamil should be avoided in patient with hypotension.

### Pathogenesis of the Ebola virus infection

Human EBOV hemorrhagic fever, characterized by uncontrolled viral replication together with immune and vascular dysregulation, has a case fatality rate of up to 90% [7]. Type I alpha/beta interferons (IFN-α/β), encoded by a single IFN-β and 13 homologous IFN-α genes in humans, represent an essential element of host defense against virus infections, including the EBOV [55]. The human EBOV infection is associated with robust IFN-α production—with plasma concentrations of IFN-α that greatly (60- to 100-fold) exceed those observed in other viral infections—but limited IFN-β production [56]. The EBOV, protected from the host interferon response by its encoded VP35 and VP24 proteins [40, 57–59], produced a heavy viral load [60–62], cytopathic damages [14, 63, 64], and cytokine dysregulation in humans [65–68]. The efficient productive replication of the EBOV inside monocyte and macrophages leads to a massive release of proinflammatory cytokines/chemokines and reactive oxygen species (ROS) [13, 15, 65, 66, 69–71], which in turn leads to diffuse endothelial cell dysfunction [72–76], disseminated intravascular coagulation [77–79], and vasomotor collapse [80–82]. The infection of the antigen presenting dendritic cells [83–86] and profound bystander apoptosis of lymphocytes [63, 87–89] impairs the development of adaptive immunity [90, 91] and EBOV-specific CD8+ T [92–94], as well as CD4+ T cells [95] that are important for the clearance of, and protection from, the EBOV infection. Infected monocyte-derived dendritic cells were impaired in the secretion of pro-inflammatory cytokines, the up-regulation of co-stimulatory molecules, and the stimulation of T cells [96]. Numbers of CD4+ and CD8+ T cells are substantially reduced in fatal human and nonhuman primate (NHP) infections before death [63, 88, 97].

### Immune evasion by the glycoproteins of the Ebola virus: implications on passive immunization and vaccine development

The EBOV is able to counteract both humoral and cell-mediated immunity through its GP1,2 and sGP [11, 98]. The overexpression of mature GP1,2 on the plasma membrane results in the masking of antigenic epitopes on GP1,2 itself and the shielding of MHC-I and integrin β, leading to evasion of antiviral immunity. Steric shielding of surface epitopes by the heavily glycosylated GP impairs the recognition and killing of EBOV-infected cells by the natural killer and cytotoxic CD8+ T cell during an acute viral infection. It may also contribute to the persistent infection in the natural reservoir host to perpetuate the spread of the EBOV [99–101]. The sGP can evade host antibody-mediated response through “antigenic subversion” by eliciting non-neutralizing antibodies that cross-react with GP1,2. Thus, the massive secretion of sGP by the EBOV may prevent effective neutralization of the virus during an EBOV infection and reduce the effectiveness of vaccines that rely upon neutralizing antibody responses against GP1,2 [20, 21]. Some of the antibodies against GP1 may lead to enhancement of infectivity of the EBOV via interaction with complement component C1q, a phenomenon known as the antibody-dependent enhancement. The EBOV initiates infection by binding its GP1 to its specific human receptor sites on the surface of human cells. The interaction of C1q enhances binding between the virus-antibody complex and the C1q ligands on the cell surface, promoting interaction between the EBOV and its receptor. These infectivity-enhancing antibodies were virus species specific and were primarily correlated with immunoglobulin IgG2a and IgM levels, but not with IgG1 levels [102, 103]. The presence of infectivity-enhancing antibodies against GP1,2 in the EBOV infection raises concerns about the effectiveness of GP-based EBOV vaccines, and the use of passive prophylaxis or treatment with GP-based antibodies [104, 105].

Antibodies against GP1 of the EBOV can be neutralizing, enhancing, or non-neutralizing and non-enhancing. Neutralizing antibodies are produced in infection by the EBOV at a relatively low frequency [106]. Some anti-EBOV antibodies are known to be neutralizing in vitro but not protective in vivo, whereas other antibodies are known to be protective in animal models in vivo, but not neutralizing in vitro [107]. Investigations of anti-GP antibodies against the EBOV showed that non-neutralizing antibodies recognized GP epitopes in the sGP or non-essential mucin-like domain of GP1, while neutralizing antibodies were specific to RBD in GP1 or conformation-dependent epitopes at the base of the GP1,2 spike where GP1 meets GP2. Two neutralizing antibodies (KZ52 and JP3K11) against EBOV—that recognize conformation-dependent epitopes comprising residues in GP1 and GP2—were identified to have quite distinct mechanisms of neutralization. KZ52 is a human recombinant IgG1 neutralizing antibody derived from a human survivor of a natural EBOV infection during the 1995 outbreak in Kikwit, Democratic Republic of Congo. KZ52 has impaired recognition for the sGP and binding was dependent on the presence of GP2 residues which are not present in the sGP. KZ52 is able to inhibit cathepsin cleavage of GP1,2. JP3K11, a monkey derived neutralizing monoclonal antibody against EBOV, recognized the cleaved, fusion-active form of GP [108]. 16 F6 is a mice derived monoclonal IgG1 antibody that neutralizes Sudan EBOV by preventing the conformational changes in GP1,2 required for membrane fusion. Both 16 F6 and KZ52 recognize GP1–GP2-bridging epitopes at the base of the GP1,2 trimer, indicating that this overlapping epitope may be one of the key sites for neutralization of the EBOV, and is thus a target for immunotherapy and a key goal of vaccine design [109]. Antibody subclass may be another important factor in protection against the EBOV. IgG2 isotype may offer more effective protection against EBOV [110, 111]. Although fully protecting guinea pigs from infection, KZ52 fails to slow viral replication and protect NHPs from the EBOV infection [112]. In contrast, rVSV-EBOGP [113–116] and rChAd-EBOGP [117–120]-based vaccination have demonstrated both prophylactic and post-exposure protection in NHPs [121]. This was previously attributed to the protective action of EBOV-specific CD4+ and CD8+ T-cell response induced by these vaccines in limiting infection and the inability of KZ52 to completely block all entries of the EBOV into cells and its subsequent explosive replication [112]. rChAd-EBOGP-based vaccination is able to generate potent humoral and cell-mediated responses. Significant antibody titers are detectable at 48 weeks post vaccination [122, 123]. CD8+ cell-mediated immunity has been shown to play a critical role in protection against the EBOV infection in NHPs in rChAd-EBOGP-based vaccination [124]. On the other hand, humoral rather than the cell-mediated response contributes to protection against the EBOV infection in NHPs in rVSV-EBOGP-based vaccination [125, 126].

Candidate vaccines expressing the EBOV GP or NP protect rodents and NHPs from the lethal EBOV infection [127–129]. Humoral and cell-mediated immune responses are working together to provide protection against the lethal EBOV infection. Either response alone may be able to limit virus replication but both arms of the immune response are required to clear the infection [97, 130]. VP proteins (VP24, VP30, VP35, and VP40) are poor inducers of cell-mediated immunity and are inaccessible to the protective effect of VP-induced neutralizing antibodies because they are not found on the surface of virions or infected cells [131]. However, the genetic sites of these internal proteins are susceptible to siRNA and PMO interference. TKM-Ebola (a siRNA targeting L-polymerase, VP24, and VP35) can be administered intravenously or subcutaneously in a lyophilized lipid nanoparticle formulation. TKM-Ebola offers post-exposure protection against the EBOV infection in NHPs. The FDA has approved an “expanded access” program for the use of TKM-Ebola in patients with confirmed or suspected infections [132, 133]. Anti-sense phosphorodiamidate morpholino oligomers AVI-6002 effectively reduce viral load, diminish virally-induced pathology, and improve survival of NHPs with the EBOV infection by targeting VP24 and VP35 mRNA. Through judicious placement of positive charges on the drug backbone, the drug is able to bind to a negative charge on the virus even if binding at one or more drug-virus base pairs are lost through mutation. This integration of dual targeting and charge complementation significantly lowers the likelihood of drug resistance through viral mutagenesis [134, 135].

### Available drugs that target the different steps of the Ebola virus life cycle

Currently available therapeutic agents that are effective in targeting the EBOV infection in cell or animal studies may include convalescent plasma, favipiravir, chloroquine, amiodarone, dronedarone, verapamil, clomiphene, toremifene, IFN-β, Na^+^/K^+^ exchangers, Na^+^/K^+^-ATPase pump inhibitors, and antioxidants. Except for convalescent plasma and favipiravir, most of the therapeutic agents under review are acting against the non-mutable targets of the host cells which participate in the replication cycle of the EBOV. They may also have a complementary role to conventional therapy in the management of the current EBOV outbreak in West African countries (see Table 1).

#### (1) Convalescent blood serum

The WHO issued a consensus statement that the use of whole blood therapies and convalescent blood serum needs to be considered as a matter of priority in the recent EBOV outbreak in West African countries [2]. The development of neutralizing antibodies and T-cell responses are important for recovery from the EBOV infection [97, 136]. Patients who are able to mount an immune response to the EBOV will begin to recover in seven to ten days and start a period of prolonged convalescence [137]. In survivors, early and increasing levels of IgG, directed mainly against the NP and the VP40, were followed by the clearance of circulating viral antigen and activation of cytotoxic T cells. In contrast, fatal infection was characterized by impaired humoral responses, with absent specific IgG and barely detectable IgM [63]. Convalescent blood has been shown to improve survival of EBOV-infected patients during the outbreak in Kikwit in 1995 [138]. Immunity against EBOV GP is sufficient to protect individuals against infection, and several vaccines based on EBOV GP are under development including recombinant adenovirus, parainfluenza virus, Venezuelan equine encephalitis virus, vesicular stomatitis virus, and virus-like particles [139]. Neutralizing human monoclonal antibodies is able to protect mouse and guinea pigs from lethal EBOV. However, the protection was achieved only by treatment shortly before or after viral infection [140–142]. The EBOV can rapidly mutate to produce antibody-escape mutants. Hence, antibody therapy may require hyperimmune polyclonal serum or a panel of monoclonal antibodies of different epitope specificities to be successful [143, 144]. These studies have laid the foundation for subsequent clinical research on the development of monoclonal antibodies [145–148] and utilization of a monoclonal antibody cocktail such as MB-003 [149], ZMAb [150], and ZMapp [151] in the treatment of the EBOV infection in NHPs. It is interesting to note that all three monoclonal antibody cocktails include one antibody that binds to or close to the glycan cap and that two of the three monoclonal antibody cocktails include at least one antibody that binds the GP1/GP2 interface, indicating that these two regions may be especially important in protection against EBOV [148]. The treatment window of monoclonal antibody therapy can be extended by the co-administration of adenovirus-vectored interferon therapy. In a guinea pig model, monoclonal antibodies combined with adenovirus-vectored interferon given three days after infection resulted in 100% survival, a significant improvement over either treatment alone [152]. A subsequent study showed that such a combination therapy is capable of saving 100% of EBOV-infected NHPs when initiated after the presence of detectable viremia along with symptoms [153].

#### (2) Favipiravir (T-705; 6-fluoro-3-hydroxy-2-pyrazinecarboxamide)

Favipiravir is a broad-spectrum inhibitor of viral RNA polymerase that is able to inhibit the replication of many RNA viruses. It is registered in Japan for the treatment of influenza virus infection [154, 155]. Favipiravir is able to suppress the replication of the EBOV in cell culture. Favipiravir, initiated at day 6 after EBOV infection, induced rapid virus clearance, reduced the biochemical parameters of disease severity, and prevented a lethal outcome in 100% of mice lacking the Type I interferon receptor [156]. Oral favipiravir taken twice daily for 14 days is able to give 100% protection against an aerosol EBOV infection in an immune-deficient mice model [157, 158]. The survival benefit was suboptimal in NHPs. Only one of the six animals tested survived. Studies using dosages that are two to five times higher and have duration longer than shown in influenza studies are being conducted for the human EBOV infection [5]. BCX4430, a synthetic adenosine analogue with a viral RNA polymerase inhibitor function, is active against the EBOV and Marburg virus in rodent and cell culture. BCX4430 completely protects NHPs from the Marburg virus infection when administered as late as 48 hours after infection [159, 160].

#### (3) Chloroquine

The antimalarial drug chloroquine is able to increase endosomal pH. An acidic endosomal environment is important for the pH-dependent activation of cysteine proteases CatB and CatL, the proteases responsible for the cleavage of EBOV GP1,2 essential for endosomal virus-host membrane fusion [35, 39, 161–163]. However, proteolytic processing of the EBOV glycoprotein has been demonstrated to be not critical for EBOV replication in cell culture [164] or NHPs [165]. A recent study using a CatB and CatL deficient mouse model for the study of the EBOV infection demonstrates that CatB and CatL activity is not absolutely required for EBOV replication. The EBOV glycoprotein cleavage seems to be mediated through a broader spectrum of proteases making therapeutic approaches targeting limited proteases unlikely to be beneficial to combat EBOV infections [166]. A broad-spectrum small molecule that targets the CatL cleavage of the EBOV and inhibits the entry of a wide variety of viruses has recently been identified. It has been examined for the potential to develop into a potent broad-spectrum antiviral medication [167].

#### (4) Cationic amphiphiles

Multiple cationic amphiphiles including amiodarone, dronedarone, verapamil, clomiphene, and toremifene have been identified as potent inhibitors of the entry of the EBOV in an NPC1-dependent fashion [38, 168]. Amiodarone used for the treatment of atrial fibrillation and ventricular cardiac arrhythmia can induce lipidosis with features similar to Niemann-Pick C disease [169]. Amiodarone and dronedarone, having basic pKa and high water solubility at acidic pH, accumulates within late endosomal compartments, blocking fluid-phase endocytosis, proteolysis and lipid trafficking, and inducing a Niemann-Pick C-like phenotype. In contrast to the Niemann-Pick type-C disease, they are not alleviated by cholesterol removal [170, 171].

Amiodarone, at concentrations that are routinely reached in human serum during anti-arrhythmic therapy (1.5–2.5 μg/ml), is a potent inhibitor of filovirus cell entry through late endosomes (IC50 0.25 μg/ml for EBOV), when induced as a Niemann-Pick C-like phenotype. Significant inhibition is observed in most endothelial and epithelial cells (e.g. macrophage, monocyte, vascular endothelial cell), except for primary hepatocyte and fibroblast. The inhibitory effect of amiodarone on the entry of the EBOV was dose-dependent and reversible upon removal of the drug. Prolonged exposure to amiodarone will not lead to a compensatory change in the host cell. A similar inhibitory property is observed with the amiodarone-related agent dronedarone and the L-type calcium channel blocker verapamil [38, 168, 172, 173].

Both clomiphene and toremifene have anti-EBOV activity in both the Vero E6 (interferon-deficient African green monkey kidney epithelial cells) and HepG2 (human hepatocellular carcinoma) cell lines. The anti-EBOV activity of clomiphene and toremifene is dependent not on its estrogen receptor antagonistic action but upon the ability of both drugs to induce a Niemann-Pick C-like phenotype to inhibit viral entry at late endosome. Clomiphene and toremifene do not disrupt the interaction between primed GP1 and NPC1, but mediate the entry block indirectly through NPC1 by targeting other endosomal/lysosomal proteins involved in the cholesterol uptake pathway whose functions may be regulated by NPC1. Clomiphene and toremifene at 60 mg/kg every other day have been shown to result in a 90% and 50% survival rate, respectively, in EBOV-infected mice compared with 100% mortality in the control group in an in vivo murine EBOV infection model. They are effective in both male and female mice [38, 174]. However, the therapeutic dose against EBOV cannot be achieved with the oral clomiphene dose used for inducing ovulation in humans [175–177]. The therapeutic dose against EBOV with tolerable side effects can be achieved with toremifene at an oral dose used in the human trial for the treatment of advanced carcinoma of the breast [178–181]. Toremifene is well absorbed and >99.5% bound to plasma protein. Toremifene undergoes extensive liver metabolism and enterohepatic recirculation. The majority of the toremifene dose is excreted as metabolites in feces. The long terminal half-life of oral toremifene may be due to both plasma protein binding and enterohepatic recirculation [182, 183].

#### (5) Interferon-beta

Interferon-induced transmembrane proteins (IFITMs) are expressed basally in the absence of IFN induction in both primary tissues and cell lines [184]. An IFITM is able to inhibit the entry of viruses to the host cell cytoplasm; permit endocytosis, but prevent subsequent viral fusion; and release viral contents into the cytosol. The human IFITM locus is located on chromosome 11 and composed of four functional genes: IFITM1, IFITM2, IFITM3, and IFITM5. IFITM4p is a pseudogene. Viruses that are restricted by IFITM proteins tend to fuse with host cell membranes in a late endosome or lysosome that precedes the induction of Type I IFN in infected cells. Viral escape from restriction by IFITM proteins could be more challenging than for antagonizing inhibitory factors that function at later stages of the virus life cycle because the opportunity for de novo synthesis of viral inhibitors is not available. All four human IFITM proteins are induced robustly by both Type I and Type II IFNs. IFITM1 is active against multiple viruses, including the EBOV and hepatitis C viruses [185–187]. IFN-β is able to induce interferon-inducible transmembrane protein production to restrict entry of the EBOV [188]. Early post-exposure treatment with IFN-β significantly increased survival time of rhesus macaques infected with a lethal dose of the EBOV, although IFN-β alone failed to alter the mortality rate. IFN-β treatment was associated with a trend towards lower plasma and tissue viral burden and pro-inflammatory cytokines production [56].

#### (6) Na^+^/K^+^ exchangers (amiloride and its derivatives)

Amiloride and its derivatives are used as potassium-sparing diuretics to treat hypertension and congestive heart failure. Apart from inhibiting epithelial Na^+^ channel and cellular Na^+^/K^+^ exchangers, these drugs could also affect the function of other less well-defined ion-exchangers (Na^+^/Ca^2+^ and Na^+^/Mg^2+^), and disturb the equilibrium of other ions, such as Mn^2+^[189–192]. The entry of the EBOV into host cells is the first step of infection and a crucial determinant of pathogenicity. Upon receptor binding between GP1 and host TIM-1 receptors, the EBOV is internalized into endosomes primarily via the macropinocytic pathway. Amiloride is able to inhibit the uptake of many viruses that utilize the macropinocytic pathway for host cell entry [193–196]. Amiloride at non-cytotoxic dosages leads to potent dose-dependent inhibition of the entry and infection of the EBOV [197, 198]. Amiloride can lead to dose-dependent inhibition of RNA synthesis. This may be due to a direct blockage of a nucleotide entry tunnel or catalytic site, or due to its effect on the equilibrium of Mg^2+^ and Mn^2+^ that are essential co-factors for polymerase activity and nucleotide insertion [199, 200]. These novel antiviral mechanisms of amiloride may uncover new targets for drug discovery against the EBOV.

#### (7) Na^+^/K^+^-ATPase pump inhibitors (ouabain, digoxin, and digitoxin)

Adenosine triphosphate (ATP) is essential in multiple steps in the replication cycle of many viruses. Na^+^/K^+^-ATPase pump is located in the plasma membrane of all animal cells to maintain the cell membrane potential. Budding of enveloped viruses is a complex phenomenon that requires concerted actions of many viral and host components. ATP may affect multiple steps in the budding process [201]. ATP is required for the assembly and maturation of a number of enveloped viruses such as the influenza virus, vaccinia virus, retrovirus, and herpes simplex virus. The Na^+^/K^+^-ATPase pump inhibitors, ouabain, Lanatoside C, strophanthidin, and digoxin are able to inhibit the replication of the influenza virus, Newcastle disease virus, and vesicular stomatitis virus through an interferon-independent mechanism [202]. Digoxin and Lanatoside C have been shown to inhibit vaccinia virus replication at non-cytotoxic doses [203]. Ouabain has shown antiviral activity against the influenza virus [204], herpes simplex virus [205], Sendai virus [206], murine leukemic virus [207], cytomegalovirus porcine reproductive and respiratory syndrome virus [208], and human cytomegalovirus virus [209]. One common feature shared by these viruses is that they all possess a lipid envelope. The EBOV is an enveloped filamentous RNA virus. The secondary matrix protein VP24—apart from its role in the evasion of host immune response, nucleocapsid formation, and regulation of replication—has an important role in viral budding and egress. Na^+^/K^+^-ATPase ATP1A1 is detected to have a close interaction with VP24 of EBOV during replication. Ouabain, at a non-cytotoxic concentration of 20nM, is able to suppress the replication of the EBOV in human MRC-5 cells [210, 211]. Among the three cardiac glycosides that may include digoxin, digitoxin, and ouabain, only digoxin is commonly used in clinical practice. Ouabain, because of its poor oral availability, is used primarily as a research tool. Further research should be conducted to investigate whether digoxin and other Na^+^/K^+^-ATPase inhibitors might play a role in the management of the EBOV or other enveloped virus infections.

#### (8) Antioxidants

The virus-associated glycoprotein GP1,2 is responsible for the activation of human macrophages [13]. The highly glycosylated mucin-like region of the GP1 subunit of GP1,2 is cytotoxic to the host cells [14]. The mucin-like region in GP1 leads to an accumulation of GP1,2 at the endoplasmic reticulum, induces endoplasmic reticulum stress [212], and activates nuclear factor kappa B (NF-κB) [213]. Mutations of the EBOV that lead to an enhanced accumulation of GP1,2 in the endoplasmic reticulum were significantly more cytotoxic than wild-type virus [214]. In human cells, the accumulation of protein in the endoplasmic reticulum will lead to endoplasmic reticulum overload response (ER-overload) which activates NF-κB through the production of ROS [215]. As a major transcription factor for antiviral and immune stimulatory activities, NF-κB is thought to play an important role in the induction of pro-inflammatory molecules, such as interleukin-1β (IL-1β), and tumor necrosis factor α (TNF-α), upon cellular responses against a virus infection [216]. The cytokine dysregulation of the EBOV involves massive ROS, NF-κB, TNF-α, and IL-1β activation [65, 66]. The effectiveness of antioxidant therapy for the EBOV infection indicates the importance of ROS in the pathogenesis of the EBOV [217]. The activation of NF-κB by ER-overload is ROS-dependent [218]. NF-κB-induced cytokine dysregulation of novel H1N1 pneumonia has been shown to be suppressible by high-dose N-acetylcysteine (NAC) antioxidant therapy at 100 mg/kg continuous infusion daily [219]. Given the poor oral availability of NAC in the range of 6% to 10% in humans [220], a therapeutic dose of NAC equivalent to the intravenous route can hardly be delivered by oral preparation. NAC is a category B drug for pregnancy and is affordable, with a wide therapeutic window. NAC has an established safety profile even in high doses and prolonged use in humans [221–223].

Cytokine dysregulation is a common feature in the EBOV infection and is associated with an enhanced mortality [65–68]. Antiviral medications directed against the mutable viral determinants of the EBOV cannot directly prevent cytokine dysregulation. The early endothelial vascular damage characteristic of the EBOV infection is not a direct effect of virus-induced cytolysis of endothelial cells, but is due to cytokine dysregulation resulting from massive release of proinflammatory cytokines/chemokines and ROS by infected macrophage and monocytes [70–72]. Lymphocytes are resistant to the EBOV infection. Cytokine dysregulation may also contribute to the diffuse bystander apoptosis of lymphocytes [63, 87–89]. With the safety profile of NAC, if the therapeutic efficacy of a high-dose NAC antioxidant therapy to manage EBOV-induced cytokine dysregulation is confirmed, it may revamp the future management of the EBOV infection.

### Proposed prophylactic and therapeutic regimen against the Ebola virus infection

There is a desperate need for a viable treatment regimen in Africa to engender hope and encourage people with symptoms and their close contacts to seek medical treatment, so as to limit the spread of the disease. This also helps to recruit and maintain adequate medical staff who are at high risk of contracting the disease. A proposed regimen against the human EBOV infection based on available medications and information from in vivo animal testing and in vitro cell culture is attached (see Tables 2 and 3). This regimen contains a cocktail of currently available medications that can target the different steps in the replication cycle of the EBOV aiming to suppress viral proliferation. It has been shown that viral load is major contribution to survival in both human and animal studies [60–62, 136]. Through viral load suppression, we may be able to prolong a patient’s survival in order to allow the development of natural body immune defense against the EBOV.

**Table 2:** **Proposed therapeutic regimen for the prophylaxes and treatment of human EBOV infection based on available therapeutic medications and information from in vivo animal testing and in vitro cell culture**

Therapeutic regimen based on available medications for ebola virus prophylaxes and treatment	
Ebola virus	Available medications
Prophylaxis^1^	Amiodarone (macrophage, monocyte & endothelial cell)
Post Needle Stick Injury Prophylaxis	IFN-β + amiodarone (macrophage, monocyte & endothelial cell) + toremifene (liver)^2,3^ + favipiravir^4^ ± convalescent blood serum
Treatment	Amiodarone (macrophage, monocyte & endothelial cell) + toremifene (liver)^2^^,3^ + favipiravir^4^ + high dose N-acetylcysteine infusion^5^ + convalescent blood serum + supportive care

^1^1 ml of blood may contain 10 ^9^ to 10 ^10^ virions in terminally ill patient. Prophylactic amiodarone therapy may protect macrophage, monocyte and endothelial cells immediately from EBOV during needle stick injury and accidental exposure and allow time for the consideration of IFN-β, toremifene, favipiravir and convalescent blood serum therapy.

^2^Amiodarone is unable to protect hepatocyte from EBOV infection.

^3^Both amiodarone and toremifene can increase the risk of QT prolongation and *Torsades de pointes.*

^4^The recommended dosage for treatment of human EBOV infection may be 2 to 5 times higher than influenza studies. Please confirm the recommended dose with the drug company.

^5^N-acetylcysteine intravenous infusion at 100 mg/kg/day to control cytokine dysregulation (e.g. add 5 g of intravenous preparation of N-acetylcysteine into each liter of intravenous replacement fluid).

**Table 3:** **Prophylaxis regimen for healthcare worker after needle stick injury**

Regimen	Oral^1^	Intravenous^4^
Central venous line	Not required	Required
Interferon-beta	6 million international units (MIU) prefilled pen via intramuscular injection (IMI) weekly for 3 weeks.^2^	6 MIU intravenous infusion over 2 hour daily for up to 3 weeks^3^ or 6 MIU prefilled pen IMI weekly for 3 weeks.
Amiodarone	600 mg p.o. twice daily for 8 days (loading) then maintenance 600 mg p.o. daily for further 3 weeks.	150 mg into 100 ml D5 over 10 minutes followed by 360 mg infusion over 6 hours then 540 mg infusion over 18 hours D1.^4^ Amiodarone 720 mg infusion daily or 600 mg p.o. twice daily for further 7 days followed by 600 mg p.o. maintenance daily for further 3 weeks.
Toremifene	800 mg p.o. on Day 1 (loading) then 400 mg p.o. daily.^5^	800 mg p.o. on Day 1 (loading) then 400 mg p.o. daily.^5^
Favipiravir	1800 mg p.o. twice daily on Day 1 (loading doses) then 800 mg p.o. twice daily.^6^	1800 mg p.o. twice daily on Day 1 (loading doses) then 800 mg p.o. twice daily.

^1^Oral regimen are for those workers who are already on amiodarone prophylaxis with a loading dose of amiodarone 600 mg p.o. twice daily for 8 days followed by maintenance amiodarone 600 mg p.o. daily. Electrocardiogram and thyroid function should be monitored.

^2^Monitor for side effect of thrombocytopenia and proteinuria.

^3^Intravenous dosage of IFN-β that are used for human hepatitis C virus infection to induce IFITM1 to limit viral entry.

^4^Intravenous regimen is for those workers who have not been on amiodarone prophylaxis and agreed for the insertion of a central venous line for drug administration. Intravenous amiodarone should be administered via central venous line to avoid phlebitis. The dosage for treatment of frequently recurring ventricular fibrillation and hemodynamically unstable ventricular tachycardia is recommended because it can achieve therapeutic drug level immediately after the first dose of amiodarone.

^5^http://www.pulmcrit.org/2014/08/could-estrogen-receptor-antagonists.html.

^6^Dosage for the treatment of human influenza virus infection in human Phase 3 trial of Favipiravir (FAVOR Study). http://www.clinicaltrials.gov/show/NCT02008344.

The recommended dosage for treatment of human EBOV infection may be 2 to 5 times higher than influenza studies. Please confirm the recommended dose with the drug company.

The EBOV has undergone a rapid mutation during its spread through humans [224–226]. The EBOV is an RNA virus the replication of which is highly error prone with nearly one viral mutation occurring during each cycle of replication. This extremely high mutation rate leads to significant genetic and antigenic diversity that allows the EBOV population to evolve resistance to antiviral medications and vaccines [227, 228]. A combination therapy has been used in the treatment of RNA virus infections, such as the human immunodeficiency virus (HIV) [229, 230] and hepatitis C [231, 232] to minimize the development of drug resistance. Given the broad cell tropism and high replication rate of the EBOV due to the potent suppression of both innate and adaptive immune responses of the host, patients with the EBOV infection have an extremely high viral load. The selective pressure in the presence of the high mutation rate and viral load during the human EBOV infection make the evolution of the EBOV viral strains resistant to a single drug inevitable. The currently available medications in the proposed regimen—which is a treatment regimen containing a cocktail of antiviral medications targeting the different steps of the EBOV replication in order to achieve maximal suppression of viral replication and to prevent the rapid development of resistance to favipiravir, the only drug in the regimen that is directed against a mutable target of the EBOV—has been shown to reduce the replication of the EBOV. [233–235].

The current EBOV vaccine (rVSV-EBOGP and rChAd-EBOGP) and therapeutic agents (ZMapp, TKM-Ebola, PMO AVI-6002, and favipiravir) under development are directed against the mutable targets of the EBOV, and their effectiveness is limited by viral mutation. The EBOV, being a RNA virus with limited coding capacity, has utilized the host’s unique metabolic pathway for its viral entry, replication, and egress. Most of the therapeutic agents in this current review are directed against non-mutable targets of the host which is independent of viral mutation. These medications are FDA-approved for the treatment of other diseases. They are available and stockpileable for immediate use. They may also have a complementary role to those therapeutic agents under development that are directed against the mutable targets of the EBOV.

The primary target of the EBOV is the mononuclear phagocytic system. The spectrum of target cells increases to include endothelial cells, fibroblasts, hepatocytes, and many other cells during the advanced stage of the disease [6, 236, 237]. The EBOV may produce a viral load of up to 10^10^ virions per ml serum in terminally ill patients [80]. Oral amiodarone prophylaxis, by inducing a Niemann-Pick C-like phenotype in the cells of the mononuclear phagocytic system, may prevent viral entry into these cells during needle stick injury. Through protection of the mononuclear system by our prophylaxis and cocktail therapy, we hope to offer a better chance of survival to these patients by allowing them to develop a natural body immune defense against the EBOV infection. The liver, containing the largest number of fixed tissue macrophages (Kupffer cells), as part of the reticuloendothelial immune defense system of the body, is a major target for the EBOV infection [238, 239]. The EBOV replicates to high titer in the liver [240]. Hepatic apoptosis may play a role in the pathogenesis of the EBOV infection [88]. Toremifene is added to the treatment regimen for hepatic protection because amiodarone does not exert inhibitory action against the EBOV in hepatocyte. However, both amiodarone and toremifene can increase QTc and the risk of Torsades de pointes. Therefore electrocardiogram should be carefully monitored if both drugs are to be used. Amiodarone, favipiravir, and toremifene are available and stockpileable in oral preparations. These properties are advantageous in outbreak situations and contingency planning of a potential EBOV epidemic or pandemic. The avoidance of intravenous administration will prevent needle stick injury in healthcare workers caring for the infected patients.

IFN-β may have potential as an adjunctive post-exposure therapy for high-risk exposure, such as needle stick injury, by inducing IFITM1 to limit entry of the EBOV. Post-exposure IFN-β treatment was associated with a trend towards lower plasma and tissue viral burden and pro-inflammatory cytokines production [56]. The reduction in viral load and cytokine dysregulation coupled with optimal supportive therapy may improve the chance of survival of the host to allow the development of natural immunity to control the underlying EBOV infection. IFITM1 is active against multiple viruses, including the EBOV [185, 188] and hepatitis C [186, 187, 241, 242]. Interferon induced IFITM1 plays an important role in the treatment of human HCV infection by inhibiting entry of HCV into the host cell [243]. Six million international units (MIU) of IFN-β intravenous administration is as effective as a three MIU twice-daily regimen for treatment of the HCV infection [244], but has lesser side effects that require discontinuation of the medication [245, 246]. As the aim of IFN-β therapy in our regimen for post needle stick prophylaxis against the EBOV infection is to induce IFITM1 to limit viral entry, the dose of IFN-β for the post needle stick prophylaxis [247, 248] or induction therapy [249, 250] for HCV infection in humans is chosen. Once infection is fully established, IFN-β are replaced by convalescent blood serum and high-dose NAC infusion for providing passive humoral immunity and for the control of ROS-dependent NF-κB-induced cytokine dysregulation respectively.

## Summary

The EBOV is classified as biosafety level 4 pathogen and is classified by Centers for Disease Control and Prevention as a category A agent of bioterrorism with no approved therapies and vaccines for its treatment but carrying a high potential for large-scale dissemination. Recent political, economic, military, and religious turbulence around the world raises concerns that the EBOV might be used as an agent of bioterrorism [251–253]. The recent EBOV epidemic is spiraling out of control in West Africa. The containment measures that worked in the past, such as isolating those who are infected and tracing their contacts, have failed due to an exponential rise in infected patients. Although the short-term (three- and six-week) probability of international spread outside the African region is small, the risk of the extension of the outbreak to other African countries followed by international dissemination on a longer time scale is not negligible, indicating that this public health emergency has the potential to grow to extraordinarily destructive dimensions [254, 255]. Although several promising therapeutic agents and vaccines against the EBOV are undergoing the Phase I human trial, the current epidemic might be outpacing the speed at which drugs and vaccines can be produced [5]. To combat such an unprecedented global public-health crisis before these experimental agents are available, alternative available interventions capable of managing the enhanced viral replication and cytokine dysregulation of the human EBOV infection should be explored and stockpiled as contingency preparation for the worst-case scenario of an impending human EBOV pandemic [256].

Like all viruses, the EBOV largely relies on host cell factors and physiological processes for its entry, replication, and egress which, in turn, lead to cytopathic damage, cytokine dysregulation, and death of the host. These non-mutable key steps inside the host may be novel targets for future therapeutic strategies against these rapidly mutating viruses. If the efficacy of amiloride, digoxin, amiodarone, and high-dose NAC antioxidant therapy against the human EBOV infection is confirmed, the availability and affordability of these stockpileable agents make them ideal medications in pandemic situation and in countries with limited resources. They may have a complementary role to other antiviral medications to prevent the emergence of resistant strains. This may also signify a major breakthrough in future management of the EBOV infection.

## Electronic supplementary material

Additional file 1:Multilingual abstracts in the six official working languages of the United Nations.(PDF 204 KB)

## References

[1] World Health Organization Ebola response roadmap Situation reports: 1 October 2014. http://apps.who.int/iris/bitstream/10665/144117/1/roadmapsitrep_21Nov2014_eng.pdf?ua=1 (Accessed 22/11/2014)

[2] World Health Organization Statement on the WHO Consultation on potential Ebola therapies and vaccines. http://www.who.int/mediacentre/news/statements/2014/ebola-therapies-consultation/en/ (Accessed 2/10/2014)

[3] World Health Organization: Ethical considerations for use of unregistered interventions for Ebola viral disease. http://apps.who.int/iris/bitstream/10665/130997/1/WHO_HIS_KER_GHE_14.1_eng.pdf (Accessed 2/10/2014)

[4] Choi JH, Croyle MA (2013). Emerging targets and novel approaches to Ebola virus prophylaxis and treatment. BioDrugs.

[5] World Health Organization: potential Ebola therapies and vaccines. http://www.who.int/csr/disease/ebola/ebola-new-interventions-02-sep-2014.pdf (Accessed 2/10/2014)

[6] Ansari AA: Clinical features and pathobiology of Ebolavirus infection. J Autoimmun. In press 10.1016/j.jaut.2014.09.001 25260583

[7] Hoenen T, Groseth A, Falzarano D, Feldmann H (2006). Ebola virus: unravelling pathogenesis to combat a deadly disease. Trends Mol Med.

[8] Volchkova VA, Klenk HD, Volchkov VE (1999). Delta-peptide is the carboxy-terminal cleavage fragment of the nonstructural small glycoprotein sGP of Ebola virus. Virology.

[9] Feldmann H, Volchkov VE, Volchkova VA, Klenk HD (1999). The glycoproteins of Marburg and Ebola virus and their potential roles in pathogenesis. Arch Virol Suppl.

[10] Volchkov VE, Volchkova VA, Slenczka W, Klenk HD, Feldmann H (1998). Release of viral glycoproteins during Ebola virus infection. Virology.

[11] Cook JD, Lee JE (2013). The secret life of viral entry glycoproteins: moonlighting in immune evasion. PLoS Pathog.

[12] Takada A, Kawaoka Y (2001). The pathogenesis of Ebola hemorrhagic fever. Trends Microbiol.

[13] Wahl-Jensen V, Kurz SK, Hazelton PR, Schnittler HJ, Ströher U, Burton DR, Feldmann H (2005). Role of Ebola virus secreted glycoproteins and virus-like particles in activation of human macrophages. J Virol.

[14] Yang ZY, Duckers HJ, Sullivan NJ, Sanchez A, Nabel EG, Nabel GJ (2000). Identification of the Ebola virus glycoprotein as the main viral determinant of vascular cell cytotoxicity and injury. Nat Med.

[15] Escudero-Pérez B, Volchkova VA, Dolnik O, Lawrence P, Volchkov VE (2014). Shed GP of Ebola Virus Triggers Immune Activation and Increased Vascular Permeability. PLoS Pathog.

[16] Lopez LA, Yang SJ, Hauser H, Exline CM, Haworth KG, Oldenburg J, Cannon PM (2010). Ebola virus glycoprotein counteracts BST-2/Tetherin restriction in a sequence-independent manner that does not require tetherin surface removal. J Virol.

[17] Lopez LA, Yang SJ, Exline CM, Rengarajan S, Haworth KG, Cannon PM (2012). Anti-tetherin activities of HIV-1 Vpu and Ebola virus glycoprotein do not involve removal of tetherin from lipid rafts. J Virol.

[18] Yasuda J (2012). Ebolavirus Replication and Tetherin/BST-2. Front Microbiol.

[19] Volchkova VA, Feldmann H, Klenk HD, Volchkov VE (1998). The nonstructural small glycoprotein sGP of Ebola virus is secreted as an antiparallel-orientated homodimer. Virology.

[20] Mohan GS, Li W, Ye L, Compans RW, Yang C (2012). Antigenic subversion: a novel mechanism of host immune evasion by Ebola virus. PLoS Pathog.

[21] Basler CF (2013). A novel mechanism of immune evasion mediated by Ebola virus soluble glycoprotein. Expert Rev Anti Infect Ther.

[22] Yang Z, Delgado R, Xu L, Todd RF, Nabel EG, Sanchez A, Nabel GJ (1998). Distinct cellular interactions of secreted and transmembrane Ebola virus glycoproteins. Science.

[23] Kindzelskii AL, Yang Z, Nabel GJ, Todd RF, Petty HR (2000). Ebola virus secretory glycoprotein (sGP) diminishes Fc gamma RIIIB-to-CR3 proximity on neutrophils. J Immunol.

[24] Wahl-Jensen VM, Afanasieva TA, Seebach J, Ströher U, Feldmann H, Schnittler HJ (2005). Effects of Ebola virus glycoproteins on endothelial cell activation and barrier function. J Virol.

[25] Alazard-Dany N, Volchkova V, Reynard O, Carbonnelle C, Dolnik O, Ottmann M, Khromykh A, Volchkov VE (2006). Ebola virus glycoprotein GP is not cytotoxic when expressed constitutively at a moderate level. J Gen Virol.

[26] Radoshitzky SR, Warfield KL, Chi X, Dong L, Kota K, Bradfute SB, Gearhart JD, Retterer C, Kranzusch PJ, Misasi JN, Hogenbirk MA, Wahl-Jensen V, Volchkov VE, Cunningham JM, Jahrling PB, Aman MJ, Bavari S, Farzan M, Kuhn JH (2011). Ebolavirus delta-peptide immunoadhesins inhibit marburgvirus and ebolavirus cell entry. J Virol.

[27] Mehedi M, Falzarano D, Seebach J, Hu X, Carpenter MS, Schnittler HJ, Feldmann H (2011). A new Ebola virus nonstructural glycoprotein expressed through RNA editing. J Virol.

[28] Aleksandrowicz P, Marzi A, Biedenkopf N, Beimforde N, Becker S, Hoenen T, Feldmann H, Schnittler HJ (2011). Ebola virus enters host cells by macropinocytosis and clathrin-mediated endocytosis. J Infect Dis.

[29] Mulherkar N, Raaben M, de la Torre JC, Whelan SP, Chandran K (2011). The Ebola virus glycoprotein mediates entry via a non-classical dynamin-dependent macropinocytic pathway. Virology.

[30] Nanbo A, Imai M, Watanabe S, Noda T, Takahashi K, Neumann G, Halfmann P, Kawaoka Y (2010). Ebolavirus is internalized into host cells via macropinocytosis in a viral glycoprotein-dependent manner. PLoS Pathog.

[31] Carette JE, Raaben M, Wong AC, Herbert AS, Obernosterer G, Mulherkar N, Kuehne AI, Kranzusch PJ, Griffin AM, Ruthel G, Dal Cin P, Dye JM, Whelan SP, Chandran K, Brummelkamp TR (2011). Ebola virus entry requires the cholesterol transporter Niemann-Pick C1. Nature.

[32] Côté M, Misasi J, Ren T, Bruchez A, Lee K, Filone CM, Hensley L, Li Q, Ory D, Chandran K, Cunningham J (2011). Small molecule inhibitors reveal Niemann-Pick C1 is essential for Ebola virus infection. Nature.

[33] Miller EH, Obernosterer G, Raaben M, Herbert AS, Deffieu MS, Krishnan A, Ndungo E, Sandesara RG, Carette JE, Kuehne AI, Ruthel G, Pfeffer SR, Dye JM, Whelan SP, Brummelkamp TR, Chandran K (2012). Ebola virus entry requires the host-programmed recognition of an intracellular receptor. EMBO J.

[34] Krishnan A, Miller EH, Herbert AS, Ng M, Ndungo E, Whelan SP, Dye JM, Chandran K (2012). Niemann-Pick C1 (NPC1)/NPC1-like1 chimeras define sequences critical for NPC1’s function as a flovirus entry receptor. Viruses.

[35] Hofmann-Winkler H, Kaup F, Pöhlmann S (2012). Host cell factors in filovirus entry: novel players, new insights. Viruses.

[36] White JM, Schornberg KL (2012). A new player in the puzzle of filovirus entry. Nat Rev Microbiol.

[37] Martinez O, Ndungo E, Tantral L, Miller EH, Leung LW, Chandran K, Basler CF (2013). A mutation in the Ebola virus envelope glycoprotein restricts viral entry in a host species- and cell-type-specific manner. J Virol.

[38] Shoemaker CJ, Schornberg KL, Delos SE, Scully C, Pajouhesh H, Olinger GG, Johansen LM, White JM (2013). Multiple cationic amphiphiles induce a Niemann-Pick C phenotype and inhibit Ebola virus entry and infection. PLoS One.

[39] Hunt CL, Lennemann NJ, Maury W (2012). Filovirus entry: a novelty in the viral fusion world. Viruses.

[40] Kühl A, Pöhlmann S (2012). How Ebola virus counters the interferon system. Zoonoses Public Health.

[41] Noda T, Kolesnikova L, Becker S, Kawaoka Y (2011). The importance of the NP: VP35 ratio in Ebola virus nucleocapsid formation. J Infect Dis.

[42] Mateo M, Carbonnelle C, Martinez MJ, Reynard O, Page A, Volchkova VA, Volchkov VE (2011). Knockdown of Ebola virus VP24 impairs viral nucleocapsid assembly and prevents virus replication. J Infect Dis.

[43] Huang Y, Xu L, Sun Y, Nabel GJ (2002). The assembly of Ebola virus nucleocapsid requires virion-associated proteins 35 and 24 and posttranslational modification of nucleoprotein. Mol Cell.

[44] Groseth A, Charton JE, Sauerborn M, Feldmann F, Jones SM, Hoenen T, Feldmann H (2009). The Ebola virus ribonucleoprotein complex: a novel VP30-L interaction identified. Virus Res.

[45] Martínez MJ, Biedenkopf N, Volchkova V, Hartlieb B, Alazard-Dany N, Reynard O, Becker S, Volchkov V (2008). Role of Ebola virus VP30 in transcription reinitiation. J Virol.

[46] Martinez MJ, Volchkova VA, Raoul H, Alazard-Dany N, Reynard O, Volchkov VE (2011). Role of VP30 phosphorylation in the Ebola virus replication cycle. J Infect Dis.

[47] Biedenkopf N, Hartlieb B, Hoenen T, Becker S (2013). Phosphorylation of Ebola virus VP30 influences the composition of the viral nucleocapsid complex: impact on viral transcription and replication. J Biol Chem.

[48] Halfmann P, Ebihara H, Marzi A, Hatta Y, Watanabe S, Suresh M, Neumann G, Feldmann H, Kawaoka Y (2009). Replication-deficient ebolavirus as a vaccine candidate. J Virol.

[49] Hoenen T, Jung S, Herwig A, Groseth A, Becker S (2010). Both matrix proteins of Ebola virus contribute to the regulation of viral genome replication and transcription. Virology.

[50] Licata JM, Johnson RF, Han Z, Harty RN (2004). Contribution of ebola virus glycoprotein, nucleoprotein, and VP24 to budding of VP40 virus-like particles. J Virol.

[51] Han Z, Boshra H, Sunyer JO, Zwiers SH, Paragas J, Harty RN (2003). Biochemical and functional characterization of the Ebola virus VP24 protein: implications for a role in virus assembly and budding. J Virol.

[52] Noda T, Ebihara H, Muramoto Y, Fujii K, Takada A, Sagara H, Hyun Kim J, Kida H, Feldmann H, Kawaoka Y (2006). Assembly and Budding of Ebolavirus. PLoS Pathog.

[53] Stahelin RV (2014). Could the Ebola virus matrix protein VP40 be a drug target?. Expert Opin Ther Targets.

[54] Soni SP, Adu-Gyamfi E, Yong SS, Jee CS, Stahelin RV (2013). The Ebola virus matrix protein deeply penetrates the plasma membrane: an important step in viral egress. Biophys J.

[55] Conzelmann KK (2005). Transcriptional activation of alpha/beta interferon genes: interference by nonsegmented negative-strand RNA viruses. J Virol.

[56] Smith LM, Hensley LE, Geisbert TW, Johnson J, Stossel A, Honko A, Yen JY, Geisbert J, Paragas J, Fritz E, Olinger G, Young HA, Rubins KH, Karp CL (2013). Interferon-β therapy prolongs survival in rhesus macaque models of Ebola and Marburg hemorrhagic fever. J Infect Dis.

[57] Basler CF, Amarasinghe GK (2009). Evasion of interferon responses by Ebola and Marburg viruses. J Interferon Cytokine Res.

[58] Ramanan P, Shabman RS, Brown CS, Amarasinghe GK, Basler CF, Leung DW (2011). Filoviral immune evasion mechanisms. Viruses.

[59] Xu W, Edwards MR, Borek DM, Feagins AR, Mittal A, Alinger JB, Berry KN, Yen B, Hamilton J, Brett TJ, Pappu RV, Leung DW, Basler CF, Amarasinghe GK (2014). Ebola Virus VP24 Targets a Unique NLS Binding Site on Karyopherin Alpha 5 to Selectively Compete with Nuclear Import of Phosphorylated STAT1. Cell Host Microbe.

[60] Bowen ETW, Baskerville A, Cantell K, Mann GF, Simpson DIH, Zuckerman AJ, Pattyn SR (1978). Ebola Virus Haemorrhagic Fever.

[61] Sanchez A, Lukwiya M, Bausch D, Mahanty S, Sanchez AJ, Wagoner KD, Rollin PE (2004). Analysis of human peripheral blood samples from fatal and nonfatal cases of Ebola (Sudan) hemorrhagic fever: cellular responses, virus load, and nitric oxide levels. J Virol.

[62] Towner JS, Rollin PE, Bausch DG, Sanchez A, Crary SM, Vincent M, Lee WF, Spiropoulou CF, Ksiazek TG, Lukwiya M, Kaducu F, Downing R, Nichol ST (2004). Rapid diagnosis of Ebola hemorrhagic fever by reverse transcription-PCR in an outbreak setting and assessment of patient viral load as a predictor of outcome. J Virol.

[63] Baize S, Leroy EM, Georges-Courbot MC, Capron M, Lansoud-Soukate J, Debré P, Fisher-Hoch SP, McCormick JB, Georges AJ (1999). Defective humoral responses and extensive intravascular apoptosis are associated with fatal outcome in Ebola virus-infected patients. Nat Med.

[64] Sullivan N, Yang ZY, Nabel GJ (2003). Ebola virus pathogenesis: implications for vaccines and therapies. J Virol.

[65] Hensley LE, Young HA, Jahrling PB, Geisbert TW (2002). Proinflammatory response during Ebola virus infection of primate models: possible involvement of the tumor necrosis factor receptor superfamily. Immunol Lett.

[66] Harcourt BH, Sanchez A, Offermann MK (1999). Ebola virus selectively inhibits responses to interferons, but not to interleukin-1beta, in endothelial cells. J Virol.

[67] Baize S, Leroy EM, Georges AJ, Georges-Courbot MC, Capron M, Bedjabaga I, Lansoud-Soukate J, Mavoungou E (2002). Inflammatory responses in Ebola virus-infected patients. Clin Exp Immunol.

[68] Leroy EM, Baize S, Volchkov VE, Fisher-Hoch SP, Georges-Courbot MC, Lansoud-Soukate J, Capron M, Debré P, McCormick JB, Georges AJ (2000). Human asymptomatic Ebola infection and strong inflammatory response. Lancet.

[69] Ströher U, West E, Bugany H, Klenk HD, Schnittler HJ, Feldmann H (2001). Infection and activation of monocytes by Marburg and Ebola viruses. J Virol.

[70] Gupta M, Mahanty S, Ahmed R, Rollin PE (2001). Monocyte-derived human macrophages and peripheral blood mononuclear cells infected with ebola virus secrete MIP-1alpha and TNF-alpha and inhibit poly-IC-induced IFN-alpha in vitro. Virology.

[71] Villinger F, Rollin PE, Brar SS, Chikkala NF, Winter J, Sundstrom JB, Zaki SR, Swanepoel R, Ansari AA, Peters CJ (1999). Markedly elevated levels of interferon (IFN)-gamma, IFN-alpha, interleukin (IL)-2, IL-10, and tumor necrosis factor-alpha associated with fatal Ebola virus infection. J Infect Dis.

[72] Geisbert TW, Young HA, Jahrling PB, Davis KJ, Larsen T, Kagan E, Hensley LE (2003). Pathogenesis of Ebola hemorrhagic fever in primate models: evidence that hemorrhage is not a direct effect of virus-induced cytolysis of endothelial cells. Am J Pathol.

[73] Sullivan NJ, Peterson M, Yang ZY, Kong WP, Duckers H, Nabel E, Nabel GJ (2005). Ebola virus glycoprotein toxicity is mediated by a dynamin-dependent protein-trafficking pathway. J Virol.

[74] Feldmann H, Bugany H, Mahner F, Klenk HD, Drenckhahn D, Schnittler HJ (1996). Filovirus-induced endothelial leakage triggered by infected monocytes/macrophages. J Virol.

[75] Simmons G, Wool-Lewis RJ, Baribaud F, Netter RC, Bates P (2002). Ebola virus glycoproteins induce global surface protein down-modulation and loss of cell adherence. J Virol.

[76] Francica JR, Matukonis MK, Bates P (2009). Requirements for cell rounding and surface protein down-regulation by Ebola virus glycoprotein. Virology.

[77] Geisbert TW, Young HA, Jahrling PB, Davis KJ, Kagan E, Hensley LE (2003). Mechanisms underlying coagulation abnormalities in ebola hemorrhagic fever: overexpression of tissue factor in primate monocytes/macrophages is a key event. J Infect Dis.

[78] Geisbert TW, Hensley LE, Jahrling PB, Larsen T, Geisbert JB, Paragas J, Young HA, Fredeking TM, Rote WE, Vlasuk GP (2003). Treatment of Ebola virus infection with a recombinant inhibitor of factor VIIa/tissue factor: a study in rhesus monkeys. Lancet.

[79] Lee AY, Vlasuk GP (2003). Recombinant nematode anticoagulant protein c2 and other inhibitors targeting blood coagulation factor VIIa/tissue factor. J Intern Med.

[80] Zampieri CA, Sullivan NJ, Nabel GJ (2007). Immunopathology of highly virulent pathogens: insights from Ebola virus. Nat Immunol.

[81] Feldmann H, Geisbert TW (2011). Ebola haemorrhagic fever. Lancet.

[82] Bray M (2005). Pathogenesis of viral hemorrhagic fever. Curr Opin Immunol.

[83] Geisbert TW, Hensley LE, Larsen T, Young HA, Reed DS, Geisbert JB, Scott DP, Kagan E, Jahrling PB, Davis KJ (2003). Pathogenesis of Ebola hemorrhagic fever in cynomolgus macaques: evidence that dendritic cells are early and sustained targets of infection. Am J Pathol.

[84] Yen B, Mulder LC, Martinez O, Basler CF: Molecular Basis for Ebola Virus VP35 Suppression of Human Dendritic Cell Maturation. J Virol. In press 10.1128/JVI.02163-14 PMC4248944 25142601

[85] Bray M, Geisbert TW (2005). Ebola virus: the role of macrophages and dendritic cells in the pathogenesis of Ebola hemorrhagic fever. Int J Biochem Cell Biol.

[86] Martinez O, Leung LW, Basler CF (2012). The role of antigen-presenting cells in filoviral hemorrhagic fever: gaps in current knowledge. Antiviral Res.

[87] Wauquier N, Becquart P, Padilla C, Baize S, Leroy EM (2010). Human fatal zaire ebola virus infection is associated with an aberrant innate immunity and with massive lymphocyte apoptosis. PLoS Negl Trop Dis.

[88] Bradfute SB, Swanson PE, Smith MA, Watanabe E, McDunn JE, Hotchkiss RS, Bavari S (2010). Mechanisms and consequences of ebolavirus-induced lymphocyte apoptosis. J Immunol.

[89] Olejnik J, Alonso J, Schmidt KM, Yan Z, Wang W, Marzi A, Ebihara H, Yang J, Patterson JL, Ryabchikova E, Mühlberger E (2013). Ebola virus does not block apoptotic signaling pathways. J Virol.

[90] Wong G, Kobinger GP, Qiu X (2014). Characterization of host immune responses in Ebola virus infections. Expert Rev Clin Immunol.

[91] Mohamadzadeh M, Chen L, Olinger GG, Pratt WD, Schmaljohn AL (2006). Filoviruses and the balance of innate, adaptive, and inflammatory responses. Viral Immunol.

[92] Bradfute SB, Warfield KL, Bavari S (2008). Functional CD8+ T cell responses in lethal Ebola virus infection. J Immunol.

[93] Gupta M, Greer P, Mahanty S, Shieh WJ, Zaki SR, Ahmed R, Rollin PE (2005). CD8-mediated protection against Ebola virus infection is perforin dependent. J Immunol.

[94] Warfield KL, Olinger G, Deal EM, Swenson DL, Bailey M, Negley DL, Hart MK, Bavari S (2005). Induction of humoral and CD8+ T cell responses are required for protection against lethal Ebola virus infection. J Immunol.

[95] Rao M, Bray M, Alving CR, Jahrling P, Matyas GR (2002). Induction of immune responses in mice and monkeys to Ebola virus after immunization with liposome-encapsulated irradiated Ebola virus: protection in mice requires CD4(+) T cells. J Virol.

[96] Mahanty S, Hutchinson K, Agarwal S, McRae M, Rollin PE, Pulendran B (2003). Cutting edge: impairment of dendritic cells and adaptive immunity by Ebola and Lassa viruses. J Immunol.

[97] Bradfute SB, Bavari S (2011). Correlates of immunity to filovirus infection. Viruses.

[98] de La Vega MA, Wong G, Kobinger GP, Qiu X (2014). The Multiple Roles of sGP in Ebola Pathogenesis. Viral Immunol.

[99] Reynard O, Borowiak M, Volchkova VA, Delpeut S, Mateo M, Volchkov VE (2009). Ebolavirus glycoprotein GP masks both its own epitopes and the presence of cellular surface proteins. J Virol.

[100] Francica JR, Varela-Rohena A, Medvec A, Plesa G, Riley JL, Bates P (2010). Steric shielding of surface epitopes and impaired immune recognition induced by the ebola virus glycoprotein. PLoS Pathog.

[101] Dowling W, Thompson E, Badger C, Mellquist JL, Garrison AR, Smith JM, Paragas J, Hogan RJ, Schmaljohn C (2007). Influences of glycosylation on antigenicity, immunogenicity, and protective efficacy of ebola virus GP DNA vaccines. J Virol.

[102] Takada A, Kawaoka Y (2003). Antibody-dependent enhancement of viral infection: molecular mechanisms and in vivo implications. Rev Med Virol.

[103] Takada A, Watanabe S, Okazaki K, Kida H, Kawaoka Y (2001). Infectivity-enhancing antibodies to Ebola virus glycoprotein. J Virol.

[104] Takada A, Feldmann H, Ksiazek TG, Kawaoka Y (2003). Antibody-dependent enhancement of Ebola virus infection. J Virol.

[105] Takada A, Ebihara H, Feldmann H, Geisbert TW, Kawaoka Y (2007). Epitopes required for antibody-dependent enhancement of Ebola virus infection. J Infect Dis.

[106] Maruyama T, Rodriguez LL, Jahrling PB, Sanchez A, Khan AS, Nichol ST, Peters CJ, Parren PW, Burton DR (1999). Ebola virus can be effectively neutralized by antibody produced in natural human infection. J Virol.

[107] Bale S, Dias JM, Fusco ML, Hashiguchi T, Wong AC, Liu T, Keuhne AI, Li S, Woods VL, Chandran K, Dye JM, Saphire EO (2012). Structural basis for differential neutralization of ebolaviruses. Viruses.

[108] Shedlock DJ, Bailey MA, Popernack PM, Cunningham JM, Burton DR, Sullivan NJ (2010). Antibody-mediated neutralization of Ebola virus can occur by two distinct mechanisms. Virology.

[109] Dias JM, Kuehne AI, Abelson DM, Bale S, Wong AC, Halfmann P, Muhammad MA, Fusco ML, Zak SE, Kang E, Kawaoka Y, Chandran K, Dye JM, Saphire EO (2011). A shared structural solution for neutralizing ebolaviruses. Nat Struct Mol Biol.

[110] Qiu X, Alimonti JB, Melito PL, Fernando L, Ströher U, Jones SM (2011). Characterization of Zaire ebolavirus glycoprotein-specific monoclonal antibodies. Clin Immunol.

[111] Wilson JA, Hevey M, Bakken R, Guest S, Bray M, Schmaljohn AL, Hart MK (2000). Epitopes involved in antibody-mediated protection from Ebola virus. Science.

[112] Oswald WB, Geisbert TW, Davis KJ, Geisbert JB, Sullivan NJ, Jahrling PB, Parren PW, Burton DR (2007). Neutralizing antibody fails to impact the course of Ebola virus infection in monkeys. PLoS Pathog.

[113] Geisbert TW, Feldmann H (2011). Recombinant vesicular stomatitis virus-based vaccines against Ebola and Marburg virus infections. J Infect Dis.

[114] Feldmann H, Jones SM, Daddario-DiCaprio KM, Geisbert JB, Ströher U, Grolla A, Bray M, Fritz EA, Fernando L, Feldmann F, Hensley LE, Geisbert TW (2007). Effective post-exposure treatment of Ebola infection. PLoS Pathog.

[115] Geisbert TW, Daddario-Dicaprio KM, Geisbert JB, Reed DS, Feldmann F, Grolla A, Ströher U, Fritz EA, Hensley LE, Jones SM, Feldmann H (2008). Vesicular stomatitis virus-based vaccines protect nonhuman primates against aerosol challenge with Ebola and Marburg viruses. Vaccine.

[116] Geisbert TW, Daddario-Dicaprio KM, Lewis MG, Geisbert JB, Grolla A, Leung A, Paragas J, Matthias L, Smith MA, Jones SM, Hensley LE, Feldmann H, Jahrling PB (2008). Vesicular stomatitis virus-based ebola vaccine is well-tolerated and protects immunocompromised nonhuman primates. PLoS Pathog.

[117] Stanley DA, Honko AN, Asiedu C, Trefry JC, Lau-Kilby AW, Johnson JC, Hensley L, Ammendola V, Abbate A, Grazioli F, Foulds KE, Cheng C, Wang L, Donaldson MM, Colloca S, Folgori A, Roederer M, Nabel GJ, Mascola J, Nicosia A, Cortese R, Koup RA, Sullivan NJ: Chimpanzee adenovirus vaccine generates acute and durable protective immunity against ebolavirus challenge. Nat Med. In press 10.1038/nm.3702 25194571

[118] Geisbert TW, Bailey M, Hensley L, Asiedu C, Geisbert J, Stanley D, Honko A, Johnson J, Mulangu S, Pau MG, Custers J, Vellinga J, Hendriks J, Jahrling P, Roederer M, Goudsmit J, Koup R, Sullivan NJ (2011). Recombinant adenovirus serotype 26 (Ad26) and Ad35 vaccine vectors bypass immunity to Ad5 and protect nonhuman primates against ebolavirus challenge. J Virol.

[119] Hensley LE, Mulangu S, Asiedu C, Johnson J, Honko AN, Stanley D, Fabozzi G, Nichol ST, Ksiazek TG, Rollin PE, Wahl-Jensen V, Bailey M, Jahrling PB, Roederer M, Koup RA, Sullivan NJ (2010). Demonstration of cross-protective vaccine immunity against an emerging pathogenic Ebolavirus Species. PLoS Pathog.

[120] Richardson JS, Pillet S, Bello AJ, Kobinger GP (2013). Airway delivery of an adenovirus-based Ebola virus vaccine bypasses existing immunity to homologous adenovirus in nonhuman primates. J Virol.

[121] Falzarano D, Geisbert TW, Feldmann H (2011). Progress in filovirus vaccine development: evaluating the potential for clinical use. Expert Rev Vaccines.

[122] Sullivan NJ, Geisbert TW, Geisbert JB, Shedlock DJ, Xu L, Lamoreaux L, Custers JH, Popernack PM, Yang ZY, Pau MG, Roederer M, Koup RA, Goudsmit J, Jahrling PB, Nabel GJ (2006). Immune protection of nonhuman primates against Ebola virus with single low-dose adenovirus vectors encoding modified GPs. PLoS Med.

[123] Ledgerwood JE, Costner P, Desai N, Holman L, Enama ME, Yamshchikov G, Mulangu S, Hu Z, Andrews CA, Sheets RA, Koup RA, Roederer M, Bailer R, Mascola JR, Pau MG, Sullivan NJ, Goudsmit J, Nabel GJ, Graham BS, VRC 205 Study Team (2010). A replication defective recombinant Ad5 vaccine expressing Ebola virus GP is safe and immunogenic in healthy adults. Vaccine.

[124] Sullivan NJ, Hensley L, Asiedu C, Geisbert TW, Stanley D, Johnson J, Honko A, Olinger G, Bailey M, Geisbert JB, Reimann KA, Bao S, Rao S, Roederer M, Jahrling PB, Koup RA, Nabel GJ (2011). CD8+ cellular immunity mediates rAd5 vaccine protection against Ebola virus infection of nonhuman primates. Nat Med.

[125] Jones SM, Feldmann H, Ströher U, Geisbert JB, Fernando L, Grolla A, Klenk HD, Sullivan NJ, Volchkov VE, Fritz EA, Daddario KM, Hensley LE, Jahrling PB, Geisbert TW (2005). Live attenuated recombinant vaccine protects nonhuman primates against Ebola and Marburg viruses. Nat Med.

[126] Marzi A, Engelmann F, Feldmann F, Haberthur K, Shupert WL, Brining D, Scott DP, Geisbert TW, Kawaoka Y, Katze MG, Feldmann H, Messaoudi I (2013). Antibodies are necessary for rVSV/ZEBOV-GP-mediated protection against lethal Ebola virus challenge in nonhuman primates. Proc Natl Acad Sci U S A.

[127] Wilson JA, Hart MK (2001). Protection from Ebola virus mediated by cytotoxic T lymphocytes specific for the viral nucleoprotein. J Virol.

[128] Ayithan N, Bradfute SB, Anthony SM, Stuthman KS, Dye JM, Bavari S, Bray M, Ozato K (2014). Ebola virus-like particles stimulate type I interferons and proinflammatory cytokine expression through the toll-like receptor and interferon signaling pathways. J Interferon Cytokine Res.

[129] Warfield KL, Swenson DL, Olinger GG, Kalina WV, Aman MJ, Bavari S (2007). Ebola virus-like particle-based vaccine protects nonhuman primates against lethal Ebola virus challenge. J Infect Dis.

[130] Olinger GG, Bailey MA, Dye JM, Bakken R, Kuehne A, Kondig J, Wilson J, Hogan RJ, Hart MK (2005). Protective cytotoxic T-cell responses induced by venezuelan equine encephalitis virus replicons expressing Ebola virus proteins. J Virol.

[131] Wilson JA, Bray M, Bakken R, Hart MK (2001). Vaccine potential of Ebola virus VP24, VP30, VP35, and VP40 proteins. Virology.

[132] Tavakoli I, Judge A, Hensley LE, Maclachlan I (2010). Postexposure protection of non-human primates against a lethal Ebola virus challenge with RNA interference: a proof-of-concept study. Lancet.

[133] Hoenen T, Feldmann H (2014). Ebolavirus in West Africa, and the use of experimental therapies or vaccines. BMC Biol.

[134] Warfield KL, Swenson DL, Olinger GG, Nichols DK, Pratt WD, Blouch R, Stein DA, Aman MJ, Iversen PL, Bavari S (2006). Gene-specific countermeasures against Ebola virus based on antisense phosphorodiamidate morpholino oligomers. PLoS Pathog.

[135] Iversen PL, Warren TK, Wells JB, Garza NL, Mourich DV, Welch LS, Panchal RG, Bavari S (2012). Discovery and early development of AVI-7537 and AVI-7288 for the treatment of Ebola virus and Marburg virus infections. Viruses.

[136] Ksiazek TG, Rollin PE, Williams AJ, Bressler DS, Martin ML, Swanepoel R, Burt FJ, Leman PA, Khan AS, Rowe AK, Mukunu R, Sanchez A, Peters CJ (1999). Clinical virology of Ebola hemorrhagic fever (EHF): virus, virus antigen, and IgG and IgM antibody findings among EHF patients in Kikwit, Democratic Republic of the Congo, 1995. J Infect Dis.

[137] Casillas AM, Nyamathi AM, Sosa A, Wilder CL, Sands H (2003). A current review of Ebola virus: pathogenesis, clinical presentation, and diagnostic assessment. Biol Res Nurs.

[138] Mupapa K, Massamba M, Kibadi K, Kuvula K, Bwaka A, Kipasa M, Colebunders R, Muyembe-Tamfum JJ (1999). Treatment of Ebola hemorrhagic fever with blood transfusions from convalescent patients. International Scientific and Technical Committee. J Infect Dis.

[139] Konduru K, Bradfute SB, Jacques J, Manangeeswaran M, Nakamura S, Morshed S, Wood SC, Bavari S, Kaplan GG (2011). Ebola virus glycoprotein Fc fusion protein confers protection against lethal challenge in vaccinated mice. Vaccine.

[140] Gupta M, Mahanty S, Bray M, Ahmed R, Rollin PE (2001). Passive transfer of antibodies protects immunocompetent and imunodeficient mice against lethal Ebola virus infection without complete inhibition of viral replication. J Virol.

[141] Parren PW, Geisbert TW, Maruyama T, Jahrling PB, Burton DR (2002). Pre- and postexposure prophylaxis of Ebola virus infection in an animal model by passive transfer of a neutralizing human antibody. J Virol.

[142] Takada A, Ebihara H, Jones S, Feldmann H, Kawaoka Y (2007). Protective efficacy of neutralizing antibodies against Ebola virus infection. Vaccine.

[143] Takada A, Feldmann H, Stroeher U, Bray M, Watanabe S, Ito H, McGregor M, Kawaoka Y (2003). Identification of protective epitopes on ebola virus glycoprotein at the single amino acid level by using recombinant vesicular stomatitis viruses. J Virol.

[144] Shahhosseini S, Das D, Qiu X, Feldmann H, Jones SM, Suresh MR (2007). Production and characterization of monoclonal antibodies against different epitopes of Ebola virus antigens. J Virol Methods.

[145] Marzi A, Yoshida R, Miyamoto H, Ishijima M, Suzuki Y, Higuchi M, Matsuyama Y, Igarashi M, Nakayama E, Kuroda M, Saijo M, Feldmann F, Brining D, Feldmann H, Takada A (2012). Protective efficacy of neutralizing monoclonal antibodies in a nonhuman primate model of Ebola hemorrhagic fever. PLoS One.

[146] Saphire EO (2013). An update on the use of antibodies against the filoviruses. Immunotherapy.

[147] Takada A (2013). Do therapeutic antibodies hold the key to an effective treatment for Ebola hemorrhagic fever?. Immunotherapy.

[148] Audet J, Wong G, Wang H, Lu G, Gao GF, Kobinger G, Qiu X (2014). Molecular characterization of the monoclonal antibodies composing ZMAb: a protective cocktail against Ebola virus. Sci Rep.

[149] Pettitt J, Zeitlin L, Kim do H, Working C, Johnson JC, Bohorov O, Bratcher B, Hiatt E, Hume SD, Johnson AK, Morton J, Pauly MH, Whaley KJ, Ingram MF, Zovanyi A, Heinrich M, Piper A, Zelko J, Olinger GG (2013). Therapeutic intervention of Ebola virus infection in rhesus macaques with the MB-003 monoclonal antibody cocktail. Sci Transl Med.

[150] Qiu X, Audet J, Wong G, Pillet S, Bello A, Cabral T, Strong JE, Plummer F, Corbett CR, Alimonti JB, Kobinger GP (2012). Successful treatment of Ebola virus-infected cynomolgus macaques with monoclonal antibodies. Sci Transl Med.

[151] Qiu X, Wong G, Audet J, Bello A, Fernando L, Alimonti JB, Fausther-Bovendo H, Wei H, Aviles J, Hiatt E, Johnson A, Morton J, Swope K, Bohorov O, Bohorova N, Goodman C, Kim D, Pauly MH, Velasco J, Pettitt J, Olinger GG, Whaley K, Xu B, Strong JE, Zeitlin L, Kobinger GP (2014). Reversion of advanced Ebola virus disease in nonhuman primates with ZMapp. Nature.

[152] Qiu X, Wong G, Fernando L, Ennis J, Turner JD, Alimonti JB, Yao X, Kobinger GP (2013). Monoclonal antibodies combined with adenovirus-vectored interferon significantly extend the treatment window in Ebola virus-infected guinea pigs. J Virol.

[153] Qiu X, Wong G, Fernando L, Audet J, Bello A, Strong J, Alimonti JB, Kobinger GP (2013). mAbs and Ad-vectored IFN-α therapy rescue Ebola-infected nonhuman primates when administered after the detection of viremia and symptoms. Sci Transl Med.

[154] Furuta Y, Gowen BB, Takahashi K, Shiraki K, Smee DF, Barnard DL (2013). Favipiravir (T-705), a novel viral RNA polymerase inhibitor. Antiviral Res.

[155] Furuta Y, Takahashi K, Shiraki K, Sakamoto K, Smee DF, Barnard DL, Gowen BB, Julander JG, Morrey JD (2009). T-705 (favipiravir) and related compounds: Novel broad-spectrum inhibitors of RNA viral infections. Antiviral Res.

[156] Oestereich L, Lüdtke A, Wurr S, Rieger T, Muñoz-Fontela C, Günther S (2014). Successful treatment of advanced Ebola virus infection with T-705 (favipiravir) in a small animal model. Antiviral Res.

[157] Smither SJ, Eastaugh LS, Steward JA, Nelson M, Lenk RP, Lever MS (2014). Post-exposure efficacy of oral T-705 (Favipiravir) against inhalational Ebola virus infection in a mouse model. Antiviral Res.

[158] Lever MS, Piercy TJ, Steward JA, Eastaugh L, Smither SJ, Taylor C, Salguero FJ, Phillpotts RJ (2012). Lethality and pathogenesis of airborne infection with filoviruses in A129 α/β -/- interferon receptor-deficient mice. J Med Microbiol.

[159] Warren TK, Wells J, Panchal RG, Stuthman KS, Garza NL, Van Tongeren SA, Dong L, Retterer CJ, Eaton BP, Pegoraro G, Honnold S, Bantia S, Kotian P, Chen X, Taubenheim BR, Welch LS, Minning DM, Babu YS, Sheridan WP, Bavari S (2014). Protection against filovirus diseases by a novel broad-spectrum nucleoside analogue BCX4430. Nature.

[160] Vere Hodge RA: Meeting report: 27th International conference on antiviral research. Antiviral Res. In press 10.1016/j.antiviral.2014.08.009 PMC7119014 25218950

[161] Madrid PB, Chopra S, Manger ID, Gilfillan L, Keepers TR, Shurtleff AC, Green CE, Iyer LV, Dilks HH, Davey RA, Kolokoltsov AA, Carrion R, Patterson JL, Bavari S, Panchal RG, Warren TK, Wells JB, Moos WH, Burke RL, Tanga MJ (2013). A systematic screen of FDA-approved drugs for inhibitors of biological threat agents. PLoS One.

[162] Gnirss K, Kühl A, Karsten C, Glowacka I, Bertram S, Kaup F, Hofmann H, Pöhlmann S (2012). Cathepsins B and L activate Ebola but not Marburg virus glycoproteins for efficient entry into cell lines and macrophages independent of TMPRSS2 expression. Virology.

[163] Misasi J, Chandran K, Yang JY, Considine B, Filone CM, Côté M, Sullivan N, Fabozzi G, Hensley L, Cunningham J (2012). Filoviruses require endosomal cysteine proteases for entry but exhibit distinct protease preferences. J Virol.

[164] Neumann G, Feldmann H, Watanabe S, Lukashevich I, Kawaoka Y (2002). Reverse genetics demonstrates that proteolytic processing of the Ebola virus glycoprotein is not essential for replication in cell culture. J Virol.

[165] Neumann G, Geisbert TW, Ebihara H, Geisbert JB, Daddario-DiCaprio KM, Feldmann H, Kawaoka Y (2007). Proteolytic processing of the Ebola virus glycoprotein is not critical for Ebola virus replication in nonhuman primates. J Virol.

[166] Marzi A, Reinheckel T, Feldmann H (2012). Cathepsin B & L are not required for ebola virus replication. PLoS Negl Trop Dis.

[167] Elshabrawy HA, Fan J, Haddad CS, Ratia K, Broder CC, Caffrey M, Prabhakar BS (2014). Identification of a broad-spectrum antiviral small molecule against severe acute respiratory syndrome coronavirus and Ebola, Hendra, and Nipah viruses by using a novel high-throughput screening assay. J Virol.

[168] Gehring G, Rohrmann K, Atenchong N, Mittler E, Becker S, Dahlmann F, Pöhlmann S, Vondran FW, David S, Manns MP, Ciesek S, von Hahn T (2014). The clinically approved drugs amiodarone, dronedarone and verapamil inhibit filovirus cell entry. J Antimicrob Chemother.

[169] Palmeri S, Battisti C, Malandrini A, Federico A (1995). Amiodarone induced lipidosis similar to Niemann-Pick C disease. Biochemical and morphological study. Life Sci.

[170] Piccoli E, Nadai M, Caretta CM, Bergonzini V, Del Vecchio C, Ha HR, Bigler L, Dal Zoppo D, Faggin E, Pettenazzo A, Orlando R, Salata C, Calistri A, Palù G, Baritussio A (2011). Amiodarone impairs trafficking through late endosomes inducing a Niemann-Pick C-like phenotype. Biochem Pharmacol.

[171] Garver WS, Heidenreich RA (2002). The Niemann-Pick C proteins and trafficking of cholesterol through the late endosomal/lysosomal system. Curr Mol Med.

[172] Rodriguez-Lafrasse C, Rousson R, Bonnet J, Pentchev PG, Louisot P, Vanier MT (1990). Abnormal cholesterol metabolism in imipramine-treated fibroblast cultures. Similarities with Niemann-Pick type C disease. Biochim Biophys Acta.

[173] Kaufmann AM, Krise JP (2008). Niemann-Pick C1 functions in regulating lysosomal amine content. J Biol Chem.

[174] Johansen LM, Brannan JM, Delos SE, Shoemaker CJ, Stossel A, Lear C, Hoffstrom BG, Dewald LE, Schornberg KL, Scully C, Lehár J, Hensley LE, White JM, Olinger GG (2013). FDA-approved selective estrogen receptor modulators inhibit Ebola virus infection. Sci Transl Med.

[175] Could estrogen receptor antagonists treated Ebola ? Josh Farkas 8/6/2014.http://www.pulmcrit.org/2014/08/could-estrogen-receptor-antagonists.html,

[176] Young SL, Opsahl MS, Fritz MA (1999). Serum concentrations of enclomiphene and zuclomiphene across consecutive cycles of clomiphene citrate therapy in anovulatory infertile women. Fertil Steril.

[177] Ghobadi C, Amer S, Lashen H, Lennard MS, Ledger WL, Rostami-Hodjegan A (2009). Evaluation of the relationship between plasma concentrations of en- and zuclomiphene and induction of ovulation in anovulatory women being treated with clomiphene citrate. Fertil Steril.

[178] Wiebe VJ, Benz CC, Shemano I, Cadman TB, DeGregorio MW (1990). Pharmacokinetics of toremifene and its metabolites in patients with advanced breast cancer. Cancer Chemother Pharmacol.

[179] Bishop J, Murray R, Webster L, Pitt P, Stokes K, Fennessy A, Olver I, Leber G (1992). Phase I clinical and pharmacokinetics study of high-dose toremifene in postmenopausal patients with advanced breast cancer. Cancer Chemother Pharmacol.

[180] Kohler PC, Hamm JT, Wiebe VJ, DeGregorio MW, Shemano I, Tormey DC (1990). Phase I study of the tolerance and pharmacokinetics of toremifene in patients with cancer. Breast Cancer Res Treat.

[181] Goldstein SR, Siddhanti S, Ciaccia AV, Plouffe L (2000). A pharmacological review of selective oestrogen receptor modulators. Hum Reprod Update.

[182] Taras TL, Wurz GT, Linares GR, DeGregorio MW (2000). Clinical pharmacokinetics of toremifene. Clin Pharmacokinet.

[183] Gennari L, Merlotti D, Stolakis K, Nuti R (2012). Pharmacokinetic evaluation of toremifene and its clinical implications for the treatment of osteoporosis. Expert Opin Drug Metab Toxicol.

[184] Tanaka SS, Yamaguchi YL, Tsoi B, Lickert H, Tam PP (2005). IFITM/Mil/fragilis family proteins IFITM1 and IFITM3 play distinct roles in mouse primordial germ cell homing and repulsion. Dev Cell.

[185] Diamond MS, Farzan M (2013). The broad-spectrum antiviral functions of IFIT and IFITM proteins. Nat Rev Immunol.

[186] Raychoudhuri A, Shrivastava S, Steele R, Kim H, Ray R, Ray RB (2011). ISG56 and IFITM1 proteins inhibit hepatitis C virus replication. J Virol.

[187] Bhanja Chowdhury J, Shrivastava S, Steele R, Di Bisceglie AM, Ray R, Ray RB (2012). Hepatitis C virus infection modulates expression of interferon stimulatory gene IFITM1 by upregulating miR-130A. J Virol.

[188] Huang IC, Bailey CC, Weyer JL, Radoshitzky SR, Becker MM, Chiang JJ, Brass AL, Ahmed AA, Chi X, Dong L, Longobardi LE, Boltz D, Kuhn JH, Elledge SJ, Bavari S, Denison MR, Choe H, Farzan M (2011). Distinct patterns of IFITM-mediated restriction of filoviruses, SARS coronavirus, and influenza A virus. PLoS Pathog.

[189] Kleyman TR, Cragoe EJ (1988). Amiloride and its analogs as tools in the study of ion transport. J Membr Biol.

[190] Nakayama S, Nomura H (1995). Mechanisms of intracellular Mg2+ regulation affected by amiloride and ouabain in the guinea-pig taenia caeci. J Physiol.

[191] Uetani T, Matsubara T, Nomura H, Murohara T, Nakayama S (2003). Ca2 + -dependent modulation of intracellular Mg2+ concentration with amiloride and KB-R7943 in pig carotid artery. J Biol Chem.

[192] Günther T, Vormann J, Cragoe EJ (1990). Species-specific Mn2+/Mg2+ antiport from Mg2(+)-loaded erythrocytes. FEBS Lett.

[193] Kerr MC, Teasdale RD (2009). Defining macropinocytosis. Traffic.

[194] Mercer J, Helenius A (2009). Virus entry by macropinocytosis. Nat Cell Biol.

[195] Raghu H, Sharma-Walia N, Veettil MV, Sadagopan S, Chandran B (2009). Kaposi’s sarcoma-associated herpesvirus utilizes an actin polymerization-dependent macropinocytic pathway to enter human dermal microvascular endothelial and human umbilical vein endothelial cells. J Virol.

[196] Harrison DN, Gazina EV, Purcell DF, Anderson DA, Petrou S (2008). Amiloride derivatives inhibit coxsackievirus B3 RNA replication. J Virol.

[197] Saeed MF, Kolokoltsov AA, Albrecht T, Davey RA (2010). Cellular entry of ebola virus involves uptake by a macropinocytosis-like mechanism and subsequent trafficking through early and late endosomes. PLoS Pathog.

[198] Hunt CL, Kolokoltsov AA, Davey RA, Maury W (2011). The Tyro3 receptor kinase Axl enhances macropinocytosis of Zaire ebolavirus. J Virol.

[199] Levi LI, Gnädig NF, Beaucourt S, McPherson MJ, Baron B, Arnold JJ, Vignuzzi M (2010). Fidelity variants of RNA dependent RNA polymerases uncover an indirect, mutagenic activity of amiloride compounds. PLoS Pathog.

[200] Ogram SA, Boone CD, McKenna R, Flanegan JB (2014). Amiloride inhibits the initiation of Coxsackievirus and poliovirus RNA replication by inhibiting VPg uridylylation. Virology.

[201] Hui EK, Nayak DP (2001). Role of ATP in influenza virus budding. Virology.

[202] Hoffmann HH, Palese P, Shaw ML (2008). Modulation of influenza virus replication by alteration of sodium ion transport and protein kinase C activity. Antiviral Res.

[203] Deng L, Dai P, Ciro A, Smee DF, Djaballah H, Shuman S (2007). Identification of novel antipoxviral agents: mitoxantrone inhibits vaccinia virus replication by blocking virion assembly. J Virol.

[204] Katzen J, Hurtado J, Lecuona E, Sznajder JI (2014). The cardioactive glycoside ouabain inhibits influenza A viral replication. Am J Respir Crit Care Med.

[205] Dodson AW, Taylor TJ, Knipe DM, Coen DM (2007). Inhibitors of the sodium potassium ATPase that impair herpes simplex virus replication identified via a chemical screening approach. Virology.

[206] Nagai Y, Maeno K, Iinuma M, Yoshida T, Matsumoto T (1972). Inhibition of virus growth by ouabain: effect of ouabain on the growth of HVJ in chick embryo cells. J Virol.

[207] Tomita Y, Kuwata T (1978). Suppression of murine leukaemia virus production by ouabain and interferon in mouse cells. J Gen Virol.

[208] Karuppannan AK, Wu KX, Qiang J, Chu JJ, Kwang J (2012). Natural compounds inhibiting the replication of Porcine reproductive and respiratory syndrome virus. Antiviral Res.

[209] Altamirano AA, Fons MP, Russell JM, Cragoe EJ, Albrecht T (1994). Human cytomegalovirus infection increases the number of ouabain-binding sites in human fibroblasts. Virology.

[210] García-Dorival I, Wu W, Dowall S, Armstrong S, Touzelet O, Wastling J, Barr JN, Matthews D, Carroll M, Hewson R, Hiscox JA: Elucidation of the Ebola virus VP24 cellular interactome and disruption of virus biology through targeted inhibition of host cell protein function. J Proteome Res. In press 10.1021/pr500556d 25158218

[211] Valente RC, Capella LS, Monteiro RQ, Rumjanek VM, Lopes AG, Capella MA (2003). Mechanisms of ouabain toxicity. FASEB J.

[212] Bhattacharyya S, Hope TJ (2011). Full-length Ebola glycoprotein accumulates in the endoplasmic reticulum. Virol J.

[213] Martinez O, Valmas C, Basler CF (2007). Ebola virus-like particle-induced activation of NF-kappaB and Erk signaling in human dendritic cells requires the glycoprotein mucin domain. Virology.

[214] Volchkov VE, Volchkova VA, Muhlberger E, Kolesnikova LV, Weik M, Dolnik O, Klenk HD (2001). Recovery of infectious Ebola virus from complementary DNA: RNA editing of the GP gene and viral cytotoxicity. Science.

[215] Pahl HL, Baeuerle PA (1997). The ER-overload response: activation of NF-kappa B. Trends Biochem Sci.

[216] Boo KH, Yang JS (2010). Intrinsic cellular defenses against virus infection by antiviral type I interferon. Yonsei Med J.

[217] Panchal RG, Reid SP, Tran JP, Bergeron AA, Wells J, Kota KP, Aman J, Bavari S (2012). Identification of an antioxidant small-molecule with broad-spectrum antiviral activity. Antiviral Res.

[218] Pahl HL, Baeuerle PA (1996). Activation of NF-kappa B by ER stress requires both Ca2+ and reactive oxygen intermediates as messengers. FEBS Lett.

[219] Lai KY, Ng WY, Osburga Chan PK, Wong KF, Cheng F (2010). High-dose N-acetylcysteine therapy for novel H1N1 influenza pneumonia. Ann Intern Med.

[220] Borgström L, Kågedal B, Paulsen O (1986). Pharmacokinetics of N-acetylcysteine in man. Eur J Clin Pharmacol.

[221] De Flora S, Izzotti A, D’Agostini F, Balansky RM (2001). Mechanisms of N-acetylcysteine in the prevention of DNA damage and cancer, with special reference to smoking-related end-points. Carcinogenesis.

[222] McElhatton PR, Sullivan FM, Volans GN (1997). Paracetamol overdose in pregnancy analysis of the outcomes of 300 cases referred to the Teratology Information Service. Reprod Toxicol.

[223] Kozer E, Koren G (2001). Management of paracetamol overdose: current controversies. Drug Saf.

[224] Gire SK, Goba A, Andersen KG, Sealfon RS, Park DJ, Kanneh L, Jalloh S, Momoh M, Fullah M, Dudas G, Wohl S, Moses LM, Yozwiak NL, Winnicki S, Matranga CB, Malboeuf CM, Qu J, Gladden AD, Schaffner SF, Yang X, Jiang PP, Nekoui M, Colubri A, Coomber MR, Fonnie M, Moigboi A, Gbakie M, Kamara FK, Tucker V, Konuwa E (2014). Genomic surveillance elucidates Ebola virus origin and transmission during the 2014 outbreak. Science.

[225] Baize S, Pannetier D, Oestereich L, Rieger T, Koivogui L, Magassouba N, Soropogui B, Sow MS, Keïta S, De Clerck H, Tiffany A, Dominguez G, Loua M, Traoré A, Kolié M, Malano ER, Heleze E, Bocquin A, Mély S, Raoul H, Caro V, Cadar D, Gabriel M, Pahlmann M, Tappe D, Schmidt-Chanasit J, Impouma B, Diallo AK, Formenty P, Van Herp M (2014). Emergence of zaire ebola virus disease in Guinea — preliminary report. N Engl J Med.

[226] Check Hayden E: Ebola virus mutating rapidly as it spreads. Nature News. In press

[227] Lauring AS, Andino R (2010). Quasispecies theory and the behavior of RNA viruses. PLoS Pathog.

[228] Domingo E, Sheldon J, Perales C (2012). Viral quasispecies evolution. Microbiol Mol Biol Rev.

[229] Luo R, Piovoso MJ, Martinez-Picado J, Zurakowski R (2011). Optimal antiviral switching to minimize resistance risk in HIV therapy. PLoS One.

[230] von Kleist M, Menz S, Stocker H, Arasteh K, Schütte C, Huisinga W (2011). HIV quasispecies dynamics during pro-active treatment switching: impact on multi-drug resistance and resistance archiving in latent reservoirs. PLoS One.

[231] Gelman MA, Glenn JS: Mixing the right hepatitis C inhibitor cocktail. Trends Mol Med. In press 10.1016/j.molmed.2010.10.005 PMC3085044 21106440

[232] Poordad F, Lawitz E, Kowdley KV, Cohen DE, Podsadecki T, Siggelkow S, Heckaman M, Larsen L, Menon R, Koev G, Tripathi R, Pilot-Matias T, Bernstein B (2013). Exploratory study of oral combination antiviral therapy for hepatitis C. N Engl J Med.

[233] Domingo E (2003). Quasispecies and the development of new antiviral strategies. Prog Drug Res.

[234] Holmes EC (2003). Error thresholds and the constraints to RNA virus evolution. Trends Microbiol.

[235] Andrei G, De Clercq E (1993). Molecular approaches for the treatment of hemorrhagic fever virus infections. Antiviral Res.

[236] Baskerville A, Fisher-Hoch SP, Neild GH, Dowsett AB (1985). Ultrastructural pathology of experimental Ebola haemorrhagic fever virus infection. J Pathol.

[237] Feldmann H, Jones S, Klenk HD, Schnittler HJ (2003). Ebola virus: from discovery to vaccine. Nat Rev Immunol.

[238] Ellis DS, Bowen ET, Simpson DI, Stamford S (1978). Ebola virus: a comparison, at ultrastructural level, of the behaviour of the Sudan and Zaire strains in monkeys. Br J Exp Pathol.

[239] Baskerville A, Bowen ET, Platt GS, McArdell LB, Simpson DI (1978). The pathology of experimental Ebola virus infection in monkeys. J Pathol.

[240] Ellis DS, Simpson IH, Francis DP, Knobloch J, Bowen ET, Lolik P, Deng IM (1978). Ultrastructure of Ebola virus particles in human liver. J Clin Pathol.

[241] Lee YJ, Zhang X, Vazquez E, Shivasabesan G, Young HA, Murphy A, Wang H, Suffredini AF, Siebenlist U, Kottilil S (2014). Impaired HCV clearance in HIV/HCV coinfected subjects treated with PegIFN and RBV due to interference of IFN signaling by IFNαR2a. J Interferon Cytokine Res.

[242] Younossi ZM, Afendy A, Stepanova M, Hossain N, Younossi I, Ankrah K, Gramlich T, Baranova A (2009). Gene expression profile associated with superimposed non-alcoholic fatty liver disease and hepatic fibrosis in patients with chronic hepatitis C. Liver Int.

[243] Wilkins C, Woodward J, Lau DT, Barnes A, Joyce M, McFarlane N, McKeating JA, Tyrrell DL, Gale M (2013). IFITM1 is a tight junction protein that inhibits hepatitis C virus entry. Hepatology.

[244] Suzuki F, Chayama K, Tsubota A, Akuta N, Someya T, Kobayashi M, Suzuki Y, Saitoh S, Arase Y, Ikeda K, Kumada H (2001). Twice-daily administration of interferon-beta for chronic hepatitis C is not superior to a once-daily regimen. J Gastroenterol.

[245] Hosseini-Moghaddam SM, Mousavi A, Alavian SM (2009). Is β-interferon a promising therapeutic option for the management of hepatitis C?. J Antimicrob Chemother.

[246] Moreno-Otero R, Trapero-Marugán M, Gómez-Domínguez E, García-Buey L, Moreno-Monteagudo JA (2006). Is interferon-beta an alternative treatment for chronic hepatitis C?. World J Gastroenterol.

[247] Kogure T, Ueno Y, Kanno N, Fukushima K, Yamagiwa Y, Nagasaki F, Kakazu E, Matsuda Y, Kido O, Nakagome Y, Ninomiya M, Shimosegawa T (2006). Sustained viral response of a case of acute hepatitis C virus infection via needle-stick injury. World J Gastroenterol.

[248] Oketani M, Higashi T, Yamasaki N, Shinmyozu K, Osame M, Arima T (1999). Complete response to twice-a-day interferon-beta with standard interferon-alpha therapy in acute hepatitis C after a needle-stick. J Clin Gastroenterol.

[249] Matsui K, Iwabuchi S, Shimizu H, Yoshida A, Fujikawa T, Takatsuka K (2010). Two week induction of interferon-beta followed by pegylated interferon alpha-2b and ribavirin for chronic infection with hepatitis C. Hepatol Res.

[250] Okushin H, Morii K, Uesaka K, Yuasa S (2010). Twenty four-week peginterferon plus ribavirin after interferon-β induction for genotype 1b chronic hepatitis C. World J Hepatol.

[251] Centers for Disease Control and Prevention: Emergency Preparedness and Response for Bioterrorism Agents/Diseases. http://www.bt.cdc.gov/agent/agentlist-category.asp (Accessed 22/11/2014)

[252] Polesky A, Bhatia G (2003). Ebola hemorrhagic fever in the era of bioterrorism. Semin Respir Infect.

[253] Balali-Mood M, Moshiri M, Etemad L (2013). Medical aspects of bio-terrorism. Toxicon.

[254] Gomes MFC, Pastore y Piontti A, Rossi L, Chao D, Longini I, Halloran ME, Vespignani A: Assessing the International Spreading Risk Associated with the 2014 West African Ebola Outbreak. PLOS Currents Outbreaks. In press 10.1371/currents.outbreaks.cd818f63d40e24aef769dda7df9e0da5 PMC4169359 25642360

[255] Fisman D, Khoo E, Tuite A: Early epidemic dynamics of the West African 2014 Ebola outbreak: estimates derived with a simple two-parameter model. PLOS Currents Outbreaks. In press 10.1371/currents.outbreaks.89c0d3783f36958d96ebbae97348d571 PMC4169344 25642358

[256] Martina BE, Osterhaus AD (2009). “Filoviruses”: a real pandemic threat?. EMBO Mol Med.

