# Supplementary material for: Human Ebola virus infection in West Africa: a review of available therapeutic agents that target different steps of the life cycle of Ebola virus
Source: Infect Dis Poverty. 2014 Nov 28;3:43. doi: 10.1186/2049-9957-3-43 (PMC4334593; doi:10.1186/2049-9957-3-43)
Supplement: Supplementary file 1 — Additional file 1:Multilingual abstracts in the six official working languages of the United Nations.(PDF 204 KB) [file 40249_2014_94_MOESM1_ESM.pdf]

Translation of the abstract into the six official working languages of the United Nations

عدوى فيروس الإيبولا البشري في غرب أفريقيا: استعراض العوامل العلاجية المتوفرة التي تستهدف خطوات مختلفة من دورة حياة فيروس إيبولا

كي لاي، جوي نج، ف تشنج

ملخص

يخرج الاندلاع الأخير لوباء فيروس الإيبولا زائير البشري في غرب أفريقيا عن نطاق السيطرة. وتصل نسبة الوفاة بسبب الحمى النزفية الناتجة عن فيروس الإيبولا البشري إلى 90٪. يصنف فيروس الإيبولا في مستوى السلامة الأحيائية 4 الممرض، وفي الفئة أ للإرهاب البيولوجي من قبل مراكز السيطرة على الأمراض والوقاية منها، مع عدم وجود العلاجات واللقاحات المعتمدة المتاحة لعلاجه بعيدا عن الرعاية الداعمة. وعلى الرغم من أن العديد من العوامل العلاجية الواعدة واللقاحات ضد فيروس الإيبولا تشهد المرحلة الأولى من التجارب البشرية، فإن سرعة انتشار الوباء الحالي قد تفوق سرعة إنتاج الأدوية واللقاحات. ومثل كل الفيروسات، يعتمد فيروس الإيبولا إلى حد كبير على عوامل الخلية المضيفة والعمليات الفسيولوجية للدخول، والنسخ، والخروج. لقد قمنا بمراجعة العوامل العلاجية المتاحة حاليا التي ثبت كونها فعالة في كبح تكاثر فيروس الإيبولا في مزارع الخلايا أو الدراسات على الحيوانات. وتوجه معظم العوامل العلاجية في هذا الاستعراض ضد أهداف غير قابلة للتغيير في المضيف، والتي تكون مستقلة عن الطفرات الفيروسية. وافقت إدارة الغذاء والدواء (FDA) على هذه الأدوية لعلاج أمراض أخرى وهي متوفرة بكميات كافية للاستخدام الفوري. وقد تكون لديها أيضا دورا مكملا لتلك العوامل العلاجية قيد التطوير التي يتم توجيهها ضد الأهداف القابلة للتغيير في فيروس الإيبولا.

Translated from English version into Arabic by Mahmoud Sami, through

TRANSLATORS
WITHOUT BORDERS

西非人类伊波拉(埃博拉)病毒感染：综述针对伊波拉病毒生命周期中不同步骤及实时可用的治疗药物

KY Lai, GWY Ng, F Cheng

摘要

最近西非爆发的伊波拉(埃博拉)病毒 (EBOV) 其蔓延之快已面临失控。EBOV 出血热有高达 90%的死亡率。目前，尚未有何药物治疗或疫苗证实有效治疗尹波拉病毒，只能作支持性治疗。EBOV 被美国疾病控制中心列为生物安全级别第 4 级病毒，也被视为是生物恐怖主义的工具之一。虽然几种针对 EBOV 治疗的药物和疫苗，正分别进行第一阶段人体试验，但目前 EVOB 蔓延速度，可能超过了在那些药物和疫苗可以成功研制和生产的速度。像所有的病毒，EBOV 入侵人体细胞和在细胞内大量繁殖后，使细胞膜破裂释放新病毒继而袭击其他细胞的过程中，很大程度上依赖人体细胞因子和生理过程。我们检视过一些在细胞培养或动物研究证实有效抑制 EBOV 繁殖、现时可用的治疗药物，发现大多数的治疗药物是针对人体细胞因子和生理过程，因这些药物的疗效不受病毒快速变种的影响。这些药物是由美国食品和药物管理局（FDA）批准，并应用于治疗其他疾病。这些药物可以储存及实时使用，它们对正在研究及发展治疗伊波拉药物，可能有互补的作用。

Translated from English version into Chinese by KY Lai

Infection par le Virus Humain Ebola dans l'Ouest de l'Afrique: compte-rendu des agents thérapeutiques disponibles qui ciblent les différentes étapes du Cycle de Vie du Virus

KY Lai, GWY Ng, F Cheng

Résumé

L'apparition récente de l'épidémie due à l'Ebolavirus humain du Zaïre (EBOV) devient incontrôlable dans l'Ouest de l'Afrique. La fièvre hémorragique provoquée par l'EBOV humain a atteint un taux de mortalité de 90%. L'EBOV est classé comme agent pathogène d'un niveau de sécurité biologique 4 et il est considéré comme un agent de bioterrorisme de catégorie A par les Centres de Contrôle et Prévention des Maladies, sans thérapies homologuées ni vaccins disponibles pour son traitement, autres que des soins de soutien. Bien que plusieurs agents thérapeutiques prometteurs et vaccins contre l'EBOV soient en cours d'essais de Phase Humaine I, l'épidémie actuelle pourrait se répandre à une vitesse supérieure à celle à laquelle les médicaments et les vaccins seraient élaborés. Comme tout virus, l'EBOV mise largement sur les facteurs de cellule hôte et les processus physiologiques pour son entrée, sa reproduction et sa sortie. Nous avons examiné les agents thérapeutiques actuellement disponibles qui se sont montrés efficaces pour supprimer la prolifération de l'EBOV dans les cultures de cellules ou d'études animales. Dans cette étude, un grand nombre de ces agents thérapeutiques sont dirigés contre des cibles non mutantes de l'hôte, qui est indépendant de la mutation virale. Ces médicaments sont approuvés par la FDA (Food and Drug Administration) pour le traitement d'autres maladies. Ils sont disponibles et stockables pour un usage immédiat. Ils peuvent avoir également un rôle complémentaire envers ces agents thérapeutiques en cours d'élaboration dirigés contre les cibles mutantes de l'EBOV.

Translated from English version into French by Ode Laforge, through

TRANSLATORS
WITHOUT BORDERS

Инфекция человеческого вируса эбола в Западной Африке: обзор имеющихся лекарственных препаратов, предназначенных для лечения на определенном этапе жизненного цикла вируса эбола.

К. У. Лай, Г. В. У. Нг, Ф. Ченг

Краткий обзор

Вспышка эпидемии Заирского эболавируса (EBOV), недавно начавшаяся в Западной Африке, выходит из-под контроля. Показатель смертности от геморрагической лихорадки Заирского эболавируса достигает 90%. Центры контроля и профилактики заболеваний классифицируют Заирский эболавирус как патоген с уровнем биологической безопасности 4 и агент категории А по биотерроризму, не имеющий одобренных лекарственных препаратов и вакцин, которые можно было бы использовать наряду с поддерживающей терапией. Несмотря на то, что несколько препаратов и вакцин против Заирского эболавируса, которые дают надежду, проходят первую фазу испытаний на людях, распространение этой эпидемий, по-видимому, опережает скорость, с которой возможно производство лекарственных препаратов и вакцин. Как и все вирусы, при проникновении в клетку, репликации и выходе из клетки Заирский эболавирус в основном

полагается на факторы клеток хозяина. Мы проанализировали доступные на данный момент лекарственные препараты, которые оказались эффективными при подавлении распространения Заирского эболавируса в клеточных культурах и при проведении испытаний на животных. Основная часть лекарственных препаратов, рассматриваемых в данном обзоре, направлены против немутриующих мишеней хозяина, которые не зависят от мутации вируса. Эти препараты одобрены Управлением по контролю за продуктами и лекарствами (FDA) для лечения других заболеваний. Их запасы доступны для немедленного использования. Они могут служить дополнением к тем лекарственным препаратам, которые разрабатываются в данный момент и направлены на мутирующие мишени Заирского эболавируса.

Translated from English version into Russian by Natalia.A, through

El brote del virus del Ébola en el África occidental: análisis de los agentes terapéuticos disponibles para cada etapa del ciclo de vida del virus del Ébola

KY Lai, GWY Ng, F Cheng

Resumen

El reciente brote epidémico de la cepa Zaire del virus del Ébola (EBOV) en África occidental continúa fuera de control. La fiebre hemorrágica del EBOV tiene una tasa de mortalidad de hasta el 90%. Los Centros para el Control y Prevención de Enfermedades (CDC por sus siglas en inglés) han clasificado al EBOV como un patógeno de nivel 4 de seguridad y un agente de bioterrorismo de categoría A, para el cual no hay por ahora ni vacunas ni terapias aprobadas (salvo terapias paliativas). Actualmente se realizan ensayos clínicos en fase I (administración en humanos) con varios agentes terapéuticos y vacunas contra el EBOV, pero el ritmo actual de propagación de la epidemia sobrepasa la velocidad a la que se pueden producir las vacunas y los medicamentos.

Como con todos los virus, el proceso de entrada, réplica y liberación del EBOV depende de factores relacionados con las células huésped y sus procesos fisiológicos. Hemos examinado agentes terapéuticos que han demostrado su efectividad en la supresión de la proliferación del EBOV en cultivos celulares o en estudios en animales. La mayoría de los agentes terapéuticos aquí estudiados atacan partes no mutables del huésped, lo cual es independiente de la mutación viral. Estos medicamentos ya están aprobados por la Administración de Medicamentos y Alimentos (FDA por sus siglas en inglés) para el tratamiento de otras enfermedades, se encuentran disponibles para su almacenamiento y uso inmediato y podrían jugar un rol complementario al de los agentes terapéuticos que se están desarrollando para los blancos mutables del EBOV.

Translated from English version into Spanish by Andrés García, through

TRANSLATORS
WITHOUT BORDERS
